# Learning interpretable representations of single-cell multi-omics data with multi-output Gaussian processes

**DOI:** 10.1093/nar/gkaf630

**Published:** 2025-07-22

**Authors:** Zahra Moslehi, Sareh AmeriFar, Kevin de Azevedo, Florian Buettner

**Affiliations:** German Cancer Consortium (DKTK), partner site Frankfurt/Mainz, a partnership between DKFZ and UCT Frankfurt–Marburg, 60590 Frankfurt am Main, Germany; German Cancer Research Center (DKFZ), 69120 Heidelberg, Germany; Department of Medicine, Goethe University Frankfurt, 60590 Frankfurt am Main, Germany; German Cancer Consortium (DKTK), partner site Frankfurt/Mainz, a partnership between DKFZ and UCT Frankfurt–Marburg, 60590 Frankfurt am Main, Germany; Department of Medicine, Goethe University Frankfurt, 60590 Frankfurt am Main, Germany; Department of Informatics, Goethe University Frankfurt, 60325 Frankfurt am Main, Germany; German Cancer Consortium (DKTK), partner site Frankfurt/Mainz, a partnership between DKFZ and UCT Frankfurt–Marburg, 60590 Frankfurt am Main, Germany; German Cancer Research Center (DKFZ), 69120 Heidelberg, Germany; Department of Medicine, Goethe University Frankfurt, 60590 Frankfurt am Main, Germany; Department of Informatics, Goethe University Frankfurt, 60325 Frankfurt am Main, Germany; German Cancer Consortium (DKTK), partner site Frankfurt/Mainz, a partnership between DKFZ and UCT Frankfurt–Marburg, 60590 Frankfurt am Main, Germany; German Cancer Research Center (DKFZ), 69120 Heidelberg, Germany; Department of Medicine, Goethe University Frankfurt, 60590 Frankfurt am Main, Germany; Department of Informatics, Goethe University Frankfurt, 60325 Frankfurt am Main, Germany; Frankfurt Cancer Institute (FCI), 60596 Frankfurt am Main, Germany

## Abstract

Learning representations of single-cell genomics data is challenging due to the nonlinear and often multi-modal nature of the data on one hand and the need for interpretable representations on the other hand. Existing approaches tend to focus either on interpretability aspects via linear matrix factorization or on maximizing expressive power via neural network-based embeddings using black-box variational autoencoders or graph embedding approaches. We address this trade-off between expressive power and interpretability by introducing a novel approach that combines highly expressive representation learning via an embedding layer with interpretable multi-output Gaussian processes within a unified framework. In our model, we learn distinct representations for samples (cells) and features (genes) from multi-modal single-cell data. We demonstrate that even a few interpretable latent dimensions can effectively capture the underlying structure of the data. Our model yields interpretable relationships between groups of cells and their associated marker genes: leveraging a gene relevance map, we establish connections between cell clusters (e.g. specific cell types) and feature clusters (e.g. marker genes for those specific cell types) within the learned latent spaces of cells and features.

## Graphical Abstract

**Graphical Abstract:**
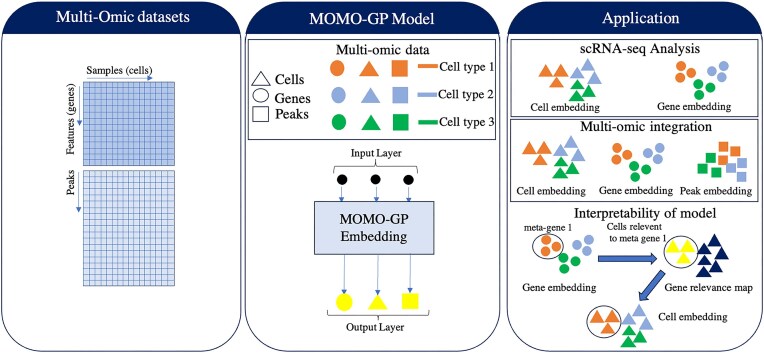

## Introduction

The single-cell genomics field has recently seen the development of many new techniques measuring different kinds of biomolecular features at the single-cell level. These methods include chromatin accessibility [1], gDNA profiling [2], methylation [3], chromatin immunoprecipitation profiling [4], protein [5], and lipid composition [6]. Each of these methods generates different data modalities that inform on different aspects of biological processes.

To integrate single-cell data from different data modalities, various computational methods have been developed. Current state-of-the-art multi-omics integration methods aim to learn a representation of cells that integrates information from all data modalities. Interpreting this new space poses a significant challenge. To relate the learned space to genes and other features, typical workflows first cluster cells in the latent space and then characterize these clusters via a differential expression analysis. Analysis on the cluster level, however, only results in a coarse-grained interpretability, omitting the structured variability within clusters [7].

Rather than conducting such post-hoc differential expression analysis on the cluster level, we propose to explicitly model the inherent correlations and dependencies between samples and genes or other features within the data.

In this paper, we introduce Multi-Omics Multi-Output Gaussian Processes (MOMO-GP) for the integration of multi-omics data. MOMO-GP embeds samples and features from different modalities (like genes and peaks) into separate interpretable latent representations. Using these representations for cells, genes, and peaks, along with gene relevance maps [8, 9] and peak relevance maps, we can directly encode cell–gene and cell–peak relations. A group of cells can be related to a group of genes and a group of peaks. Learning these three embeddings jointly helps to achieve a high expressive power for each of the embeddings, while maintaining interpretability. MOMO-GP is not restricted to just genes or peaks, and it can be used for any other view. Since MOMO-GP directly links samples and features from different modalities together via their respective embedding spaces, it facilitates clustering-free marker detection as well as the cluster-agnostic analysis of feature–feature interactions. The direct encoding of cell–feature or feature–feature relations has been previously proposed in SIMBA, where all features and samples are co-embedded into a common latent space [10]. SIMBA constructs a graph in which cells and features are represented as nodes, and relations between these entities are encoded as edges. Then, a graph embedding approach is utilized to embed all nodes into a common low-dimensional space. However, the feature embeddings learned by SIMBA only tend to have a limited expressive power, which may stem the inherent restriction to a single shared latent space between all cells and all features. Our results show that learning separate representations for features and cells substantially outperforms SIMBA, offering better expressive power and more faithful representations.

The primary concept of MOMO-GP is to learn a latent variable model that explicitly models dependencies between samples, features, and views. Standard latent variable models only model dependencies between samples and their multi-view versions between samples and views. We extend the framework of Gaussian Process Latent Variable Models (GP-LVM) [9, 11], a probabilistic kernel PCA via GP regression, which treats all features as independent. To explicitly model the dependencies between features (genes), we introduce an additional kernel to model the covariance between features. We then connect this feature kernel with the standard sample kernel that models dependencies between cells, via the Kronecker product. For modeling multi-view data, we introduce additional kernels to capture dependencies among features of each view. We employ the manifold relevance determination (MRD) approach [12] to learn for each dimension of the cell embedding, whether it is a private dimension that is specific to an individual view or whether it is shared between views and jointly models variance in multiple views.

In summary, MOMO-GP

- simultaneously learns a feature embedding for every modality and a shared cell embedding;
- is designed to find a trade-off between expressive power and interpretability by explicitly linking nonlinear dependencies between features and cells;
- outperforms other existing methods. In the sample space, it performs similar to other baseline and existing algorithms but provides better interpretability. In the feature space, our model outperforms SIMBA, the only other baseline to simultaneously learn and link feature and cell embeddings.

## Materials and methods

### Background

#### A brief review on Gaussian processes

Gaussian processes (GPs) are a type of probabilistic model that defines a distribution over functions [13]. In GPs, a function is conceptualized as an infinite-dimensional vector, where a prior distribution is established over a set of *N* instances of them. This prior distribution follows a Gaussian distribution parameterized by a mean and a covariance. The mean is typically assumed to be zero, while the covariance is determined by a function of the input space on which the process operates. The covariance quantifies the similarity between all pairs from the input space, which is modeled by the kernel function. By sampling from the GP prior distribution, when a pair of input data points are close together, their function values are highly correlated. Consequently, this process yields a smooth function over the input space. When the input space is regarded as a latent variable, it is referred to as the GP Latent Variable Model (GP-LVM) [11, 14].

A multi-output GP is an extension of the traditional single-output GP to simultaneously predict multiple correlated outputs, leveraging shared information across different tasks via a coregionalization matrix [15]. To more efficiently model the relation among different outputs and allow for new outputs at test time, in the Latent Variable Multiple Output Gaussian Processes (LV-MOGP) the coregionalization matrix is replaced by a kernel matrix [16]. LV-MOGP then infers a latent space representing the information about different outputs. The kernel of this multi-output GP is separable and can be expressed as the Kronecker product of two individual kernels. The first kernel captures the similarity between samples, in the input space, while the second kernel measures the similarity between pairs of features. Note that in contrast to the GP-LVM, inputs are observed.

#### GP Latent Variable Model

Here, we briefly explain the mathematical foundation of GP-LVM. Let the observed data in a high-dimensional space be denoted by $\mathbf {Y} \in \mathbb {R}^{I \times J}$, where *I* represents the number of samples and *J* denotes the number of features. The matrix **Y** is considered a noisy version of the true values $\mathbf {F} \in \mathbb {R}^{I \times J}$. The relationship between **Y** and **F** is described by a likelihood function. We define a nonlinear mapping between the high-dimensional data **F** and a low-dimensional latent representation $\mathbf {A}\in \mathcal {A} =\mathbb {R}^{I\times r_{1}}$, where *r*_1_ is the number of dimensions in the latent space, with *r*_1_ ≪ *J*. This mapping is governed by a GP, with **A** serving as the latent inputs.

The GP-LVM method assumes that the features are independent, while the samples exhibit strong correlations. The GP learns the correlation structure between the data points in the high-dimensional space by inferring **A** in a way that ensures a smooth mapping from the latent to the data space. This model maintains the integrity of dissimilarities, meaning that two points far apart in the data space cannot be positioned too closely in the latent space, as such proximity would imply a discontinuity in the mapping [17].

### MOMO-GP algorithm

#### Probabilistic model of MOMO-GP: single-view version

In this section, we will briefly introduce the main concept of our probabilistic model, starting with the single-view version. The detailed formulation of the model is presented in Supplementary Methods.

In single-cell RNA-seq datasets, dependencies exist between different samples as well as different features. For example, cells of a specific cell type have a high similarity, and there are dependencies among all marker genes of similar cell types. Inspired by the LV-MOGP [16] and following [18], we model these dependencies between output dimensions via a kernel matrix and introduce an additional *r*_2_-dimensional latent variable $\mathbf {B}\in \mathcal {B} =\mathbb {R}^{J\times r_{2}}$ to the standard GP-LVM in order to model the correlation structure between the genes. Note that in LV-MOGP, the inputs are observed, whereas in our model, both latent variables **A** and **B** need to be inferred.

To facilitate an efficient implementation of the model, we follow [18] and represent the observed data via a triple store where an observed training sample is represented as (*i*, *j*, *y*_*i*,*j*_), where ∀(*i*, *j*) ∈ [1, *I*] × [1, *J*] with sample *i*, feature *j*, and corresponding entries in the observed matrix *y*_*i*, *j*_. In this way, the long vectors $\mathbf {y}\in \mathbb {R}^{I \cdot J}$ are defined. Following the idea of LV-MOGP to define the dependencies of samples and features, a new coregionalization kernel needs to be defined as the Kronecker product of two individual kernels, one on the latent inputs and one on the latent outputs. Since this Kronecker product computes the correlation for all combinations of *I* samples and *J* features in matrix **F**, the size of the coregionalization kernel is (*I* · *J*) × (*I* · *J*).

We finally write our model as $p(\mathbf {f}) = \mathcal {N}\left(\mathbf {f}|\mathbf {0},\mathbf {K}^{\text{coreg}} \right)$, where $\mathbf {f} \in \mathbb {R}^{(I \cdot J)}$ and $\mathbf {K}^{\text{coreg}} \in \mathbb {R}^{(I \cdot J) \times (I \cdot J)}$, and the vector $\mathbf {y}\in \mathbb {R}^{I \cdot J}$ is defined as the noisy version of **f**. To compute this GP model, we need to compute the inverse of the covariance matrix **K**^coreg^, which in a naive implementation has a complexity of $\mathcal {O}(n^3)$, where *n* is the number of samples; in this case, *n* = *I* · *J*, where *I* is the number of samples and *J* is the number of features. In genomics data, we often have a large number of cells (*I*) as well as genes (*J*). To decrease the time complexity of the model and make the problem tractable, we employ the idea of sparse GPs. The fundamental idea behind sparse GPs is to approximate the full GP model using a smaller set of representative points known as inducing points [19]. These inducing points are significantly fewer than the original data points and effectively summarize the essential information in the data. They are selected to ensure that the GP model can be well approximated with this smaller set, allowing the model to capture the core structure of the data without considering all data points simultaneously, thereby reducing computational complexity. For this purpose, we define the variables $\mathbf {A}_{u}\in \mathcal {A} = \mathbb {R}^{m_{A} \times r_1}$ and $\mathbf {B}_{u}\in \mathcal {B} =\mathbb {R}^{m_{B} \times r_2}$. Here, **A**_*u*_ and **B**_*u*_ are inducing points in the latent spaces $\mathcal {A}$ and $\mathcal {B}$, respectively. By leveraging this concept, we can already reduce the time complexity of our model from $\mathcal {O}(I\cdot J)^{3}$ to $\mathcal {O}((I\cdot J)\cdot (m_{A}\cdot m_{B})^{2})$, where *m*_*A*_ and *m*_*B*_ are a subset of samples and features (inducing points) with *m*_*A*_ ≪ *I* and *m*_*B*_ ≪ *J*. Moreover, we enforce the same number of inducing points *m* for **A**_*u*_ and **B**_*u*_ and that allows us to replace the Kronecker product with an elementwise product, as proposed in [18]. Using this trick, we can then further reduce computational complexity to $\mathcal {O}((I\cdot J)\cdot m^{2})$. We empirically confirm this linear complexity for up to 7 000 000 entities in Fig. 10.

In our model, the variables that need to be optimized include **A**, **A**_*u*_, **B**, **B**_*u*_, and other kernel parameters. To capture the nonlinear structure of the data, we follow the approach proposed in [18] and combine an embedding layer with a GP layer. Instead of directly optimizing the variables **A** and **B**, we use an embedding function that embeds each cell and each feature into dense vectors of fixed size.

More formally, we map all indices in the range 1, ..., *I* (representing cells) and 1, ..., *J* (representing features) to matrices of size *I* × *r*_1_ and *J* × *r*_2_, respectively, using an embedding layer. Here, *r*_1_ represents the size of the input embedding space, and *r*_2_ represents the size of the output embedding space. For computing **A**_*u*_ and **B**_*u*_, we randomly select from 1, ..., *I* and 1, ..., *J*, respectively, and pass them through the embedding layer to obtain matrices **A**_*u*_ and **B**_*u*_ of size *m* × *r*_1_ and *m* × *r*_2_, respectively. During training, the weights of this embedding layer are optimized.

**Figure 1.**
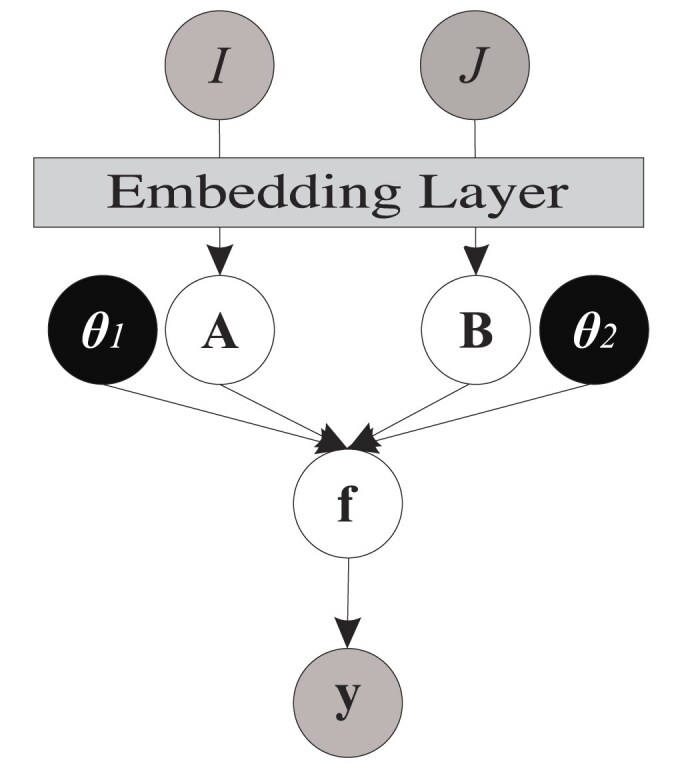
Probabilistic graphical model for single-view version of MOMO-GP. In this model, $\boldsymbol{\theta }_{1}$ and $\boldsymbol{\theta }_{2}$ are the parameters of the covariance functions $\mathbf {K}^{\mathcal {A}}$ and $\mathbf {K}^{\mathcal {B}}$.

Figure 1 provides a graphical illustration of this proposed probability model. In this graphical model, shaded and white nodes represent observed and latent variables, respectively, while black circles denote parameters that need to be optimized by deriving the likelihood function. For further information, please refer to the Supplementary data. Additionally, Algorithm S1 in Supplementary Methods outlines all the steps involved in the implementation process.

#### Probabilistic model of multi-view MOMO-GP

In the multi-view version of our method, we integrate a latent variable model that considers dependencies between samples, features, and views. This is different from standard latent variable models, which typically only model dependencies between samples or between samples and views. Similar to the single-view version, we use a kernel to model the covariance between samples. Then, we introduce additional kernels to capture dependencies among features of each view. Since the samples between different modalities are shared, we use one embedding for the samples but learn individual feature representations for each modality. Then, we link modalities via MRD [12], which aims to decompose the representation of all data views into shared and private latent spaces. In brief, using automatic relevance determination (ARD) priors [13], each view of the data is allowed to estimate a separate vector of ARD parameters. This view-wise relevance parameter allows us to learn for each dimension of the cell embedding, whether it is a private dimension that is specific to an individual modality or whether it is shared between modalities and explains variance in multiple modalities.

To illustrate how MOMO-GP can be extended to a two-view version, we consider single-cell gene expression data and single-cell ATAC-seq data.

Let *I* denote the number of samples, *J* denote the number of features for the first dataset (genes), and *K* denote the number of features for the second dataset (peaks). $\mathbf {Y}_{1} \in \mathbb {R}^{I \times J}$ and $\mathbf {Y}_{2} \in \mathbb {R}^{I \times K}$ represent our observed datasets generated from **F**_1_ and **F**_2_, respectively. However the same as single-view version, we use a triple store for the both datasets. In this way, the long vectors $\mathbf {y}_{1}\in \mathbb {R}^{I \cdot J}$ and $\mathbf {y}_{2}\in \mathbb {R}^{I \cdot K}$ are defined. These are the noisy versions of $\mathbf {f}_{1}\in \mathbb {R}^{I \cdot J}$ and $\mathbf {f}_{2}\in \mathbb {R}^{I \cdot K}$. $\mathbf {A} \in \mathbb {R}^{I \times r_{1}}$ represents the low-dimensional embedding of cells, $\mathbf {B} \in \mathbb {R}^{J \times r_{2}}$ represents the embedding of genes, and $\mathbf {C} \in \mathbb {R}^{K \times r_{3}}$ represents the embedding of peaks.

Rather than directly learning **A**, **B**, and **C**, we utilize an embedding layer to map row and column indices of the given datasets into these latent variables. We define one coregionalization kernel of size (*I* · *J*) × (*I* · *J*), formed by the Kronecker product of the covariance matrices $\mathbf {K}^{\mathcal {A}}$ and $\mathbf {K}^{\mathcal {B}}$, and another one of size (*I* · *K*) × (*I* · *K*), formed by the Kronecker product of $\mathbf {K}^{\mathcal {A}}$ and $\mathbf {K}^{\mathcal {C}}$. We then define two GPs, one for generating **f**_1_ using the first kernel and another one for generating **f**_2_ using the second kernel.

To fit the model, we define variables **A**_*u*_ and **B**_*u*_ to make the first GP sparse via inducing points, and **A**_*u*_ and **C**_*u*_ are defined similarly for the second GP. Similar to **A**, **B**, and **C**, the variables **A**_*u*_, **B**_*u*_, and **C**_*u*_ are selected from the spaces $\mathcal {A}$, $\mathcal {B}$, and $\mathcal {C}$, respectively, but their sizes are much smaller than **A**, **B**, and **C**. We choose the same number of inducing points **A**_*u*_, **B**_*u*_, and **C**_*u*_ as proposed in [18], and replace the Kronecker product with an elementwise product to further reduce the time complexity of the algorithm. For more details, refer to the Supplementary data.

In this model, the embedding of samples **A** is shared for generating both datasets **y**_1_ and **y**_2_. We utilize the idea of MRD to allocate some latent dimensions of **A** shared between both datasets and some dimensions which are private for each dataset. Specifically, our kernel of samples $\mathbf {K}^{\mathcal {A}}$ would be different for **y**_1_ and **y**_2_. $\mathbf {K}_{\mathbf {y}_{1}}^{\mathcal {A}}$ is an RBF kernel with ARD of the form


(1) \begin{eqnarray*}
k_{\mathbf {y}_{1}}^{\mathcal {A}}(\mathbf {a}_{i}, \mathbf {a}_{i^{\prime }}) = \left(\sigma _{\mathbf {y}_{1}}^{\text{ard}}\right)^{2} {\rm e}^{-\frac{1}{2} \sum _{r=1}^{r_{1}} w_{1}^{r}({a}_{i}^{r} - {a}_{i^{\prime }}^{r})^{2}},
\end{eqnarray*}

and similarly $\mathbf {K}_{\mathbf {y}_{2}}^{\mathcal {A}}$ is defined. However, we learn a common latent space for both **y**_1_ and **y**_2_, but with the help of ARD weight vectors $\mathbf {w}_{1} \in \mathbb {R}^{r_{1}}$ and $\mathbf {w}_{2} \in \mathbb {R}^{r_{1}}$, we can identify which dimensions are shared among both modalities and which dimensions are specifically assigned to each modality. In this way, the latent space **A** can be segmented as $\mathbf {A}=[\mathbf {A}_{\mathbf {y}_{1}},\mathbf {A}_{\mathbf {S}},\mathbf {A}_{\mathbf {y}_{2}}]$, in which **A**_**S**_ is shared between both datasets for the set of dimensions *r* ∈ {1, ..., *r*_1_} for which $w_{1}^{r}, w_{2}^{r} >\delta$, where *δ* is a number close to zero. The private space $\mathbf {A}_{\mathbf {y}_{1}}$ (resp. $\mathbf {A}_{\mathbf {y}_{2}}$) is defined for the set of dimensions for which $w_{1}^{r} >\delta$, $w_{2}^{r} \,<\, \delta$ (resp. $w_{2}^{r} >\delta$, $w_{1}^{r} \,<\, \delta$).

**Figure 2.**
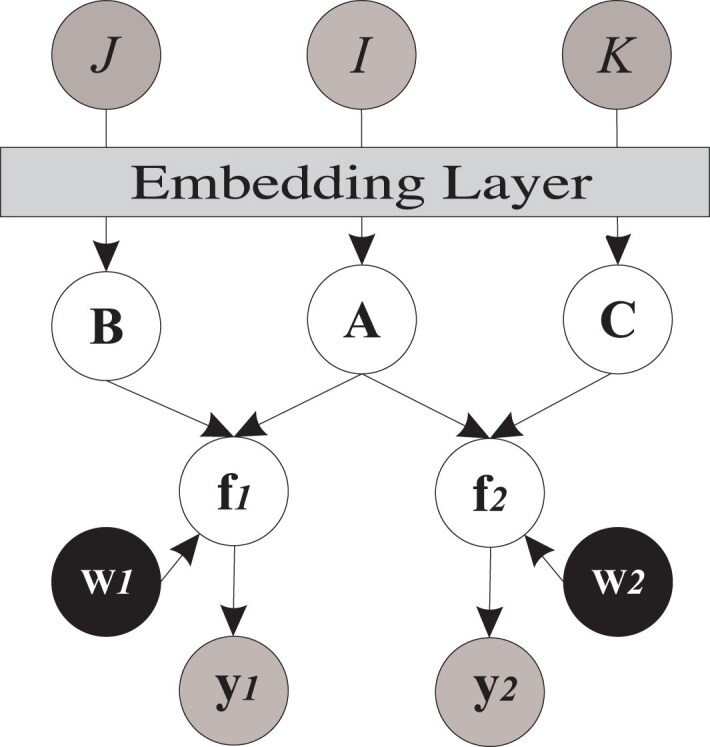
Probabilistic graphical model for multi-view version of MOMO-GP.

Figure 2 graphically illustrates this model. The training algorithm of the multi-view version is similar to Algorithm S1 in Supplementary Methods, but we consider that here we have two given datasets **y**_1_ and **y**_2_, two feature latent spaces **B** and **C**, and four sets of inducing variables, and thus two sets of **K**_*uu*_, **K**_*uf*_, and **K**_*fu*_ for generating **f**_1_ and **f**_2_. For optimizing **w**_1_ and **w**_2_, we update these ARD kernel parameters by maximizing the marginal likelihood distribution. The detailed steps involved in the implementation process are given in Algorithm S2.

#### Implementation

The MOMO-GP model was implemented in Python using the GPFlow2 [20] and GPFlux [21] packages. The code for MOMO-GP is available at https://github.com/MLO-lab/MOMO-GP.

### Evaluation on single-cell data

#### Single-cell RNA-seq and ATAC-seq integration

To evaluate our method using both RNA-seq and ATAC-seq data, we used two datasets. The first dataset is the PBMC 10k dataset from 10x Genomics, which contains single-cell multiome ATAC and gene expression data from human peripheral blood mononuclear cells (PBMCs) of a healthy donor, with granulocytes removed through cell sorting. This dataset includes 11 909 cells, 36 601 genes, and 134 726 peaks, and published on 9 September 2020 (https://support.10xgenomics.com/single-cell-multiome-atac-gex/datasets/1.0.0/pbmc_granulocyte_sorted_10k). The second dataset is a Slide-tag dataset profiling T cells and monocytes from human melanoma samples, generated using single-cell multiome ATAC and gene expression sequencing. It includes 2535 cells, 27 173 genes, and 53 451 peaks [22].

#### CITE-seq integration

We also evaluated MOMO-GP on CITE-seq data of PBMCs. CITE-seq datasets contain transcriptome-wide measurements for single cells, including gene expression data and surface protein level information for a few dozen proteins. The dataset consists of 5247 cells, 33 538 genes, and 32 proteins, and is based on the 10x Genomics 5k PBMCs from a healthy donor, prepared with a panel of TotalSeq-b antibodies using V3 chemistry and published on 29 May 2019 (https://support.10xgenomics.com/single-cell-gene-expression/datasets/3.0.2/5k_pbmc_protein_v3).

#### Data pre-processing

To preprocess the PBMC 10k and PBMC 5k (CITE-seq) datasets, we used Scanpy [23] for normalization, logarithmic transformation, clustering, and cluster annotations. Preprocessing of the Slide-tag dataset was done using Seurat, following the primary publication [24].

##### PBMC data: single-cell RNA-seq

We applied quality control by filtering low-quality cells and those with high mitochondrial content. Genes detected in only a small number of cells were excluded. After normalization and logarithmic transformation, we used Leiden clustering to annotate cell types. Clusters showing noise, high ribosomal gene expression, or proliferating cells were removed. Further feature selection was performed to retain only the most variable and biologically relevant genes for downstream analysis.

##### PBMC data: single-cell ATAC-seq

We filtered peaks detected in a minimal number of cells and retained cells with an appropriate number of accessible chromatin regions. Latent semantic indexing was used for normalization, followed by the same log-normalization approach as in single-cell RNA sequencing (scRNA-seq). Clusters were annotated based on marker genes, and additional filtering focused on the most variable chromatin regions. Only cells passing the respective quality control criteria were retained in each modality. For integration purposes, only cells present in both modalities were considered.

The number of cells in the intersection of the RNA-seq and ATAC-seq datasets, used in our analysis of the PBMC 10k dataset, amounted to 9393.

##### PBMC data: single-cell protein data

Protein expression data were normalized using the denoised and scaled by background method [25]. The dataset comprises 32 proteins, and the number of cells in the intersection of the RNA-seq and protein expression data, utilized in our analysis of the 5k PBMC CITE-seq dataset, amounted to 3891.

##### Slide-tag data

Filtering for low-quality cells was performed following the primary publication. Log-normalization was then performed on both modalities of this dataset. For analysis with MOMO-GP, we retained the 2000 most variable genes and the 5000 most variable peaks based on highest variance.

### Benchmarking

We compared our method with several commonly used baselines and related methods: Principal Component Analysis (PCA) [26], Uniform Manifold Approximation and Projection (UMAP) [27], Bayesian Gaussian Process Latent Variable Model (BGPLVM) [28], SCVI [29], and SIMBA [10].

PCA was selected as a linear dimensional reduction approach due to its ability to provide interpretable results. In our results, we ran PCA via Scanpy.

UMAP, a nonlinear manifold learning algorithm widely used for visualizing biological data points, was also run via Scanpy. However, UMAP does not provide interpretable embeddings.

MOMO-GP extends the GP-LVM by incorporating dependencies for both inputs and outputs, while GP-LVM assumes all output values are independent. We evaluate a Bayesian implementation (BGPLVM) as a nonlinear model that provides interpretable results but does not support feature embedding. We utilized the implementation of BGPLVM developed with GPytorch [30].

SCVI was selected as a state-of-the-art algorithm in neural network-based embedding algorithms. For SCVI, we employed the SCVI-tools package [29], which is designed for single-cell data and built on PyTorch and AnnData.

SIMBA is a method for co-embedding samples and features. To the best of our knowledge, it is the only method that learns and links both sample and feature embeddings in single-cell data. We compared our sample and feature embedded data with the outputs of SIMBA both qualitatively and using quantitative metrics.

### Evaluating the quality of the results

For analyzing the results, we use two different quantitative metrics.

When cell type information is available, we can use accuracy as a criterion to evaluate the results. First, we apply unsupervised clustering on the embedded data via GMM or *k*-means clustering, setting *k* to the number of cell types. To compute clustering accuracy, we assign the predicted label as the most frequent cell type of each cluster. Then, accuracy is computed by dividing the total number of data points with the correct predicted label by the total number of all data points. Formally, it is as follows:


(2) \begin{eqnarray*}
\mathrm{ACC} = \frac{\sum _{i=1}^{n} \delta (\hat{c}_{i}, c_{i})}{n} \times 100,
\end{eqnarray*}

where *n* is the number of data points, *c*_*i*_ is the correct real label, $\hat{c}_{i}$ is the predicted label, and the delta function *δ*(*s*, *t*) = 1 when *s* = *t*, otherwise it is 0 [31, 32].

The other metric used is the adjusted Rand index (ARI) [33]. ARI evaluates the similarity between two data clusterings. It considers all pairs of samples, counting those assigned to the same or different clusters in both the predicted and true clusterings. In our case, one of the clusterings would be the grouping of data points based on their cell types and the reference clustering the unsupervised clustering in the embedding space (here GMM clustering). This allows us to evaluate the discrimination of cell types provided by our learned embedding. Unlike the raw Rand index, the ARI adjusts for the chance grouping of elements, providing a more accurate assessment of clustering performance also in the case of imbalanced cluster sizes (e.g. due to the presence of rare cell types).

In our experiments, we provide the ACC and ARI values for both sample embeddings and feature embeddings.

## Results

### Single-cell RNA-seq analysis with MOMO-GP

While we propose a multi-omics algorithm with sample and feature embedding, we first verify the effectiveness of our method in the single-omics case. Specifically, we check whether our embeddings are competitive compared to popular algorithms. This initial validation ensures that our model can produce high-quality embeddings before extending its application to multi-omics data. To this end, we utilized the RNA modality of the PBMC 10k dataset to assess the performance of single-view MOMO-GP. Similarly, we used the RNA modality of the 5k PBMC CITE-seq dataset for evaluation purposes. The results are evaluated across various aspects.

#### Cell embedding

Our approach using multi-output GPs focuses on learning a low-dimensional embedding where each dimension is interpretable. Unlike linear methods, our nonlinear approach allows us to use only a handful of latent variables to model the data effectively. This results in a low-dimensional representation that maintains both interpretability and nonlinearity, providing a faithful and meaningful representation of the underlying data structure. In this section, we demonstrate that the MOMO-GP embedding of cells is comparable to or better than other existing methods.

**Figure 3.**
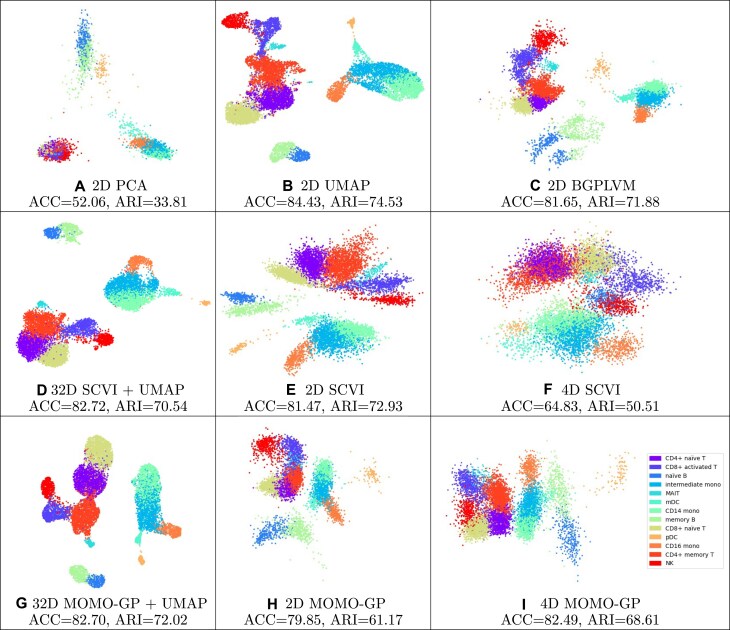
2D visualization of cells in the PBMC 10k dataset for scRNA-seq data using various methods: (**A**) 2D PCA, (**B**) 2D UMAP, (**C**) 2D BGPLVM, (**D**) 32D SCVI+UMAP, (**E**) 2D SCVI, (**F**) 4D SCVI, (**G**) 32D MOMO-GP+UMAP, (**H**) 2D MOMO-GP, and (**I**) 4D MOMO-GP.

We projected the gene expression data points into a 2D space after applying PCA [26], UMAP [27], and BGPLVM [28] algorithms. The results of the PBMC 10k dataset are depicted in Fig. 3 and Supplementary Fig. S6 (and Supplementary Fig. S1 for the PBMC 5k-CITE-seq dataset). We ran SCVI [29] and our method in three different setups: embedding data points into a 32-dimensional space and displaying the 2D visualization of UMAP embedding of projected data; 2D embedding of data; and embedding data points into a 4D space and selecting the best results of two latent factors from these four different dimensions. The results for other two latent factors from these four different dimensions are given in Supplementary Fig. S9. Data points in this figure are colored based on their cell type. After applying the MOMO-GP algorithm, the separation between the 13 different cell types, including CD4^+^ naïve T, CD8^+^ activated T, naïve B, intermediate monocytes, MAIT, mDC, CD14 monocytes, memory B, CD8^+^ naïve T, pDC, CD16 monocytes, CD4^+^ memory T, and NK cells, is well-defined, and the components appear well coordinated. For a more quantitative comparison, ACC and ARI values for different methods are also presented. To further support our conclusions, we have included additional performance metrics. Specifically, we also report results for silhouette, *k*-means ARI, *k*-means NMI, and isolated labels, which support our finding that 2D UMAP, 2D BGPLVM, 32D SCVI+UMAP, and 32D MOMOGP+UMAP exhibit comparable performance (Supplementary Figs S7 and S8; for interpretation of the additional metrics, see Supplementary data). However, MOMO-GP provides additional feature embeddings, which offer a significant advantage and are discussed in detail in the following sections.

When comparing the ARI values with respect to *k*-means and GMM as choices for unsupervised clustering, we consistently find that GMM achieves higher ARI scores in most cases. This indicates a stronger alignment between GMM clusters and class labels, which is the primary reason we report metrics with respect to GMM clustering for all our experiments in the main text.

#### Gene embedding

**Figure 4.**
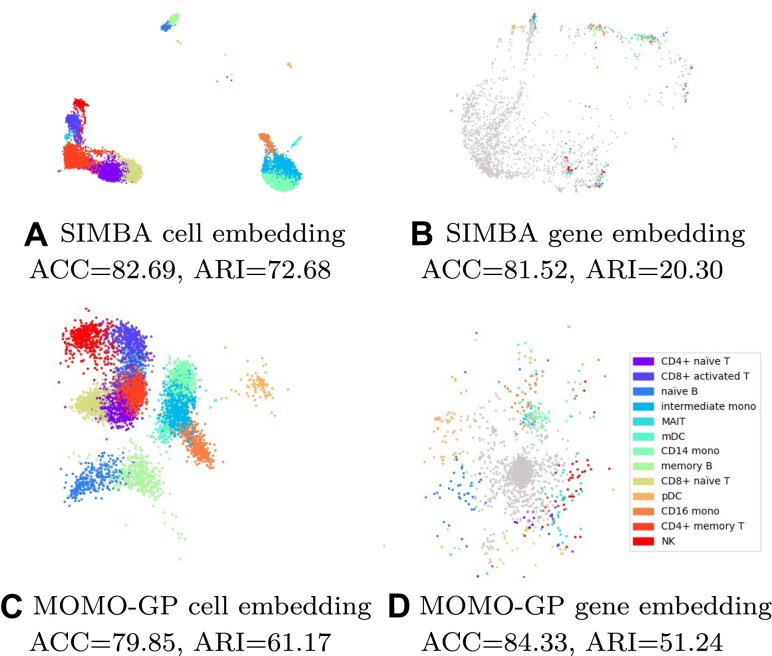
Visualization of PBMC 10k dataset using SIMBA and MOMO-GP embedding techniques for scRNA-seq data. (**A**) SIMBA–UMAP embedding of cells, with cell types color-coded, in a 50D space. (**B**) SIMBA–UMAP embedding of genes, highlighting the top 100 marker genes per cell type, color-coded by their respective cell types, in a 50D space. Non-marker genes are shown in gray. (**C**) MOMO-GP embedding of cells in a 2D space. (**D**) MOMO-GP embedding of genes in a 2D space.

In this section, we demonstrate that even with a few latent dimensions, the underlying structure of the data can be captured effectively without utilizing all genes. Specifically, we set the number of latent dimensions for both cell and gene representations to 2 and visualize the 2D embedding of all cells and genes. The results for the PBMC 10k dataset are depicted in Fig. 4 (and Supplementary Fig. S2 for the PBMC 5k-CITE-seq dataset). In these figures, cells are colored based on their cell types. Additionally, for each cell type, we identify the top 100 differentially expressed marker genes and color them according to their respective cell types. From the visualization, it is evident that our gene embedding using only two latent factors yields meaningful insights. Although there is not a perfect separation between marker genes of all different cell types, all marker genes of a specific cell type tend to form a cohesive cluster. Another interesting observation in this figure is the presence of a gray cluster in the middle of Fig. 4D. These genes do not exhibit specific biological associations with any particular cell types, leading them to form a distinct cluster within our embedding. To further elucidate the role of these genes, we selected the top 20 genes located near the center of the data, within the gray region (listed in Supplementary Table S1). These genes are characterized by their involvement in diverse regulatory processes, including immune responses, development, and gene expression. Many of these genes are long noncoding RNAs involved in gene regulation (e.g. AC022445.1, EMX2OS, AC005481.1, AC024933.1, LINC02821, CARMN, AL590999.1, AC079035.1, AL589740.1, and AC092134.1). Moreover, we report both ACC and ARI values for gene and cell embeddings. We observe that for gene embeddings, ARI values tend to be low while ACC values remain high. This discrepancy arises from the way we select features: we consider the top 100 marker genes for each cell type. As a result, many genes lack a specific class label and are grouped into the “unknown” class, which we incorporate into our computations. Since many points fall into this “unknown” class, it introduces class imbalance in the data. In imbalanced datasets, it is more common to see high ACC values but lower ARI values. This is the reason that we report both metrics alongside the visualizations, ensuring a more comprehensive and robust evaluation of our results.

Additionally, we provide cell and gene embeddings of RNA data from the PBMC 10k dataset using the SIMBA method. The default number of latent dimensions in SIMBA is set to 50. The cell and gene embeddings generated by SIMBA, followed by UMAP visualization, are presented in Fig. 4A and B, respectively. For the results of MOMO-GP with 50 latent dimensions followed by UMAP visualization, refer to Supplementary Fig. S10. While SIMBA’s cell embedding demonstrates effective separation among various cell types, its gene embedding noticeably underperforms compared to MOMO-GP.

#### Interpretability of the model

**Figure 5.**
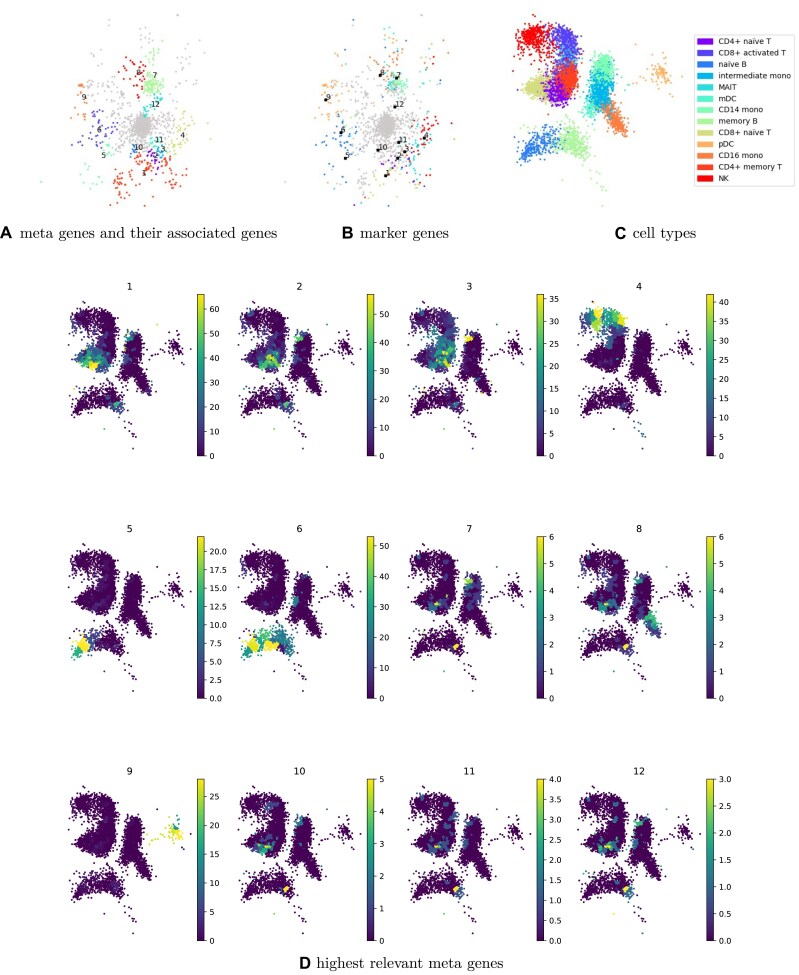
Exploration of the PBMC 10k dataset using a gene relevance map, which automatically identifies connections between groups of cells and genes. (**A**) Gene embedding colored according to genes associated with each metagene. (**B**) Gene embedding colored by marker genes specific to each cell type. (**C**) Cell embedding colored by cell types. (**D**) Gene relevance plot highlighting regions where gene contribution is highest. For instance, metagene 9 is enriched for pDC marker genes and exhibits significant relevance in the corresponding region of the cell embedding.

A significant characteristic of MOMO-GP is its capability to project both samples and features in a latent space. This feature becomes particularly valuable when we aim to establish connections between groups of samples and groups of genes in the latent space without relying on any ground truth about cell and gene labels. To achieve this, we adopt the concept of gene relevance maps [8, 9], the details of which are provided in the Supplementary data. In brief, a local gene relevance plot delineates the regions in a cell embedding where a gene’s contributions are most pronounced. In our analysis, instead of identifying the single highest relevant gene for each area, we opt to identify groups of metagenes relevant to that area. We leverage the MOMO-GP gene embedding and identify metagenes [7] (groups of similar genes) from our gene embedding. Subsequently, we link the highest globally relevant metagenes to certain cells using the concept of gene relevance maps. This approach enables us to link a group of genes (belonging to one metagene) to a group of cells. The outcomes of this experiment on the PBMC 10k dataset are depicted in Fig. 5 (and Supplementary Fig. S3 for the PBMC 5k-CITE-seq dataset). In Fig. 5A, we illustrate the gene embedding results, with all genes belonging to one metagene uniformly colored. In Fig. 5B, we define the top 100 marker genes based on cell embedding for each cell type and color them according to their corresponding cell type. For the cell embedding, we highlight the areas belonging to specific cell types by coloring all data points based on their cell type, as shown in Fig. 5C. We evaluate the relationship between cell embedding and gene embedding by doing two evaluations, one of them using the gene embedding and another one using the cell embedding: In the first evaluation, we analyze metagenes in the gene embedding space, capturing the structural organization of data points. We then consider marker genes and assess their alignment with these metagenes in gene embedding space. Table 1 presents the proportion of the majority cell type for each metagene along with *P*-values to indicate statistical significance.

**Table 1.** PBMC 10k dataset: majority cell types for each metagene based on marker genes, along with their proportion values (expressed as percentages of cell-type coverage) and *P*-values, using gene embedding

Metagene	Majority cell type (marker genes)	Cell-type coverage	*P*-value
1	CD8^+^ naïve T	39.06	2.86 × 10^−12^
2	CD4^+^ naïve T	44.44	3.22 × 10^−3^
3	CD4^+^ memory T	100.00	2.69 × 10^−6^
4	NK	61.36	1.55 × 10^−19^
5	Naïve B	76.92	1.67 × 10^−9^
6	Naïve B	78.38	1.03 × 10^−25^
7	CD14 mono	43.88	2.12 × 10^−22^
8	CD16 mono	70.00	6.30 × 10^−12^
9	pDC	100.00	2.39 × 10^−26^

In the second evaluation, we use a gene relevance map to visualize each metagene’s association with cell groups in the cell embedding space. To quantify this, we identify cells relevant to each metagene by applying a threshold on relevance scores and examining their cell types. Figure 5D delineates the areas where each metagene is relevant. For example, upon analyzing the gene relevance map for metagene 9, we observe that all cells in this area are pDC cells. The majority cell type and its proportion for each metagene are also reported in Table 2.

**Table 2.** PBMC 10k dataset: majority cell types, identified through the gene relevance map, along with their proportion values (expressed as percentages of cell-type coverage) using cell embedding

Metagene	Majority cell type (gene relevance map)	Cell-type coverage
1	CD8^+^ naïve T	49.16
2	CD4^+^ naïve T	62.37
3	CD4^+^ memory T	57.16
4	NK	65.29
5	Naïve B	97.94
6	Naïve B	67.74
7	CD14 mono	40.38
8	CD16 mono	50.21
9	pDC	94.67

For a more in-depth evaluation, we would like to understand what the metagenes are and whether they capture biologically meaningful gene sets. To do that, we employed gene set enrichment analysis [34] with over-representation analysis (hypergeometric test) [35], implemented by the Gene Set Enrichment Analysis in Python (GSEAPY) package [36]. ORA aids in identifying gene sets that are predominantly present in our gene lists of interest. For this analysis, the gene lists comprise the genes of each metagene, while the gene set is selected from the human MSigDB collections [37]. Specifically, we select the C8 cell type signature gene set for bone marrow. The outcomes of this experiment on the PBMC 10k dataset are presented in Table 3 (and Supplementary Table S2 for the PBMC 5k-CITE-seq dataset). For each metagene, we sort enriched gene sets based on the combined enrichment score (computed with GSEAPY) and show the two most strongly enriched ones that adjusted *P*-value <.05. Those metagenes that do not have any enriched gene sets are not shown in the table.

**Table 3.** PBMC 10k dataset: a list of gene sets enriched for each metagene

Metagene	Term	Adjusted *P*-value	Combined score	Cell-type coverage
1	Naïve T	2.02 × 10^−43^	2235.34	89.57
1	CD8 T	3.6 × 10^−2^	26.99	49.16
2	Naïve T	2.73 × 10^−8^	597.65	68.28
4	NK	8.38 × 10^−52^	14262.21	34.71
4	CD8 T	3.46 × 10^−3^	103.89	65.28
5	Follicular B	3.24 × 10^−6^	391.46	100
6	Follicular B	6.53 × 10^−24^	2545.37	100
6	Plasma	1.14 × 10^−3^	107.62	NA
7	Neutrophil	6.53 × 10^−24^	3013.32	NA
7	Immature neutrophil	6.55 × 10^−21^	786.68	NA
8	Monocyte	4.98 × 10^−23^	4046.71	57.38
9	Dendritic	5.81 × 10^−20^	4608.97	94.66
9	CD34 B	1.6 × 10^−2^	109.18	NA
11	CD34^+^ Multilin	0.255	444.41	NA
11	Pro-B	0.304	36.79	NA
12	CD34^+^ ERP Early	0.284	108.61	NA
12	CD34^+^ LMPP	0.304	68.24	NA

*Note*: To compute cell-type coverage for the term “naïve T,” we include all CD4^+^ naïve T and CD8^+^ naïve T cells. For the term “CD8 T,” we count only CD8^+^ naïve T cells. Due to overlaps between these two groups, the cumulative value for metagene 1 exceeds 100%.

Via the gene sets enriched in each metagene, we can approximately define the cell type associated with each group of genes. By comparing these enriched cell types with those relevant in the gene relevance map, we can validate first that MOMO-GP learns a meaningful gene embedding with similar genes being grouped together. Second, we validate that our relevance-based approach links gene and cell embeddings in a meaningful fashion. To do that, we have to identify the cells in which a metagene is relevant and check their cell types. Then, by comparing their cell types and the cell type associated with the respective metagene, we can validate that metagenes capture meaningful groups of genes. For example, according to the ORA results of metagene 1, we observe a relationship between T cells and genes of this metagene. On the other hand, via the gene relevance map, we find a relation between metagene 1 and T cells. In Table 3, we present the cell type coverage values. For each metagene, we compute its gene relevance map and compute the fraction of cells with relevance score above a threshold *τ* that match the cell type predicted by GSEA. For example, in the case of metagene 1, 89.57% of the cells with a relevance score above *τ* = 30 are classified as naïve T cells. These results highlight the strong structure within the gene-embedded data generated by MOMO-GP and its clear and meaningful relationship with cell embeddings. Furthermore, our cell and gene embeddings combined with the gene relevance map allow us to identify new or rare cell types. From the results presented in Table 3, we observe that Pro-B cells are highly enriched for genes associated with metagene 11. There is a direct link between Pro-B cells and memory B cells. Pro-B cells represent an early stage of B cell development, eventually maturing into naïve B cells and, upon antigen exposure, differentiating into memory B cells. Additionally, CD34^+^ Multilin cells are also enriched for these genes. Some CD34^+^ Multilin progenitors serve as precursors to T-cell progenitors. As illustrated in the gene relevance map in Fig. 5D, two small clusters of memory B cells and CD4^+^ naïve T cells are associated with this metagene. The same analysis can be applied to metagene 12. Based on the results in Table 3, we observe that CD34^+^ LMPP cells are enriched for metagene 12, and they play a significant role in the development of memory B cells and naïve T cells. This relationship is also reflected in the gene relevance map for this metagene. So, this approach allows us to establish a connection between newly identified genes and rare cell types.

### Single-cell multi-omics integration with MOMO-GP

To demonstrate the effectiveness of our model on multi-view data, we examine its performance on three datasets: the PBMC 10k and Slide-tag datasets, which combine paired scRNA-seq and scATAC-seq data, and the 5k PBMC CITE-seq dataset, comprising gene expression data and protein-level information. We quantify the quality of cell embeddings and feature embeddings for all modalities.

#### Cell embedding

**Figure 6.**
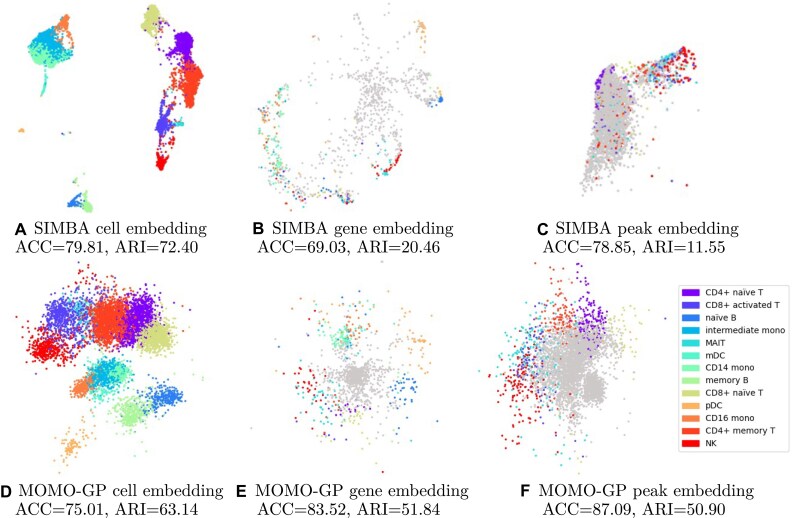
Exploration of the PBMC 10k dataset with SIMBA and MOMO-GP embedding techniques applied to both scRNA-seq and scATAC-seq data. (**A**) SIMBA–UMAP embedding of cells color-coded by cell types. (**B**) SIMBA–UMAP embedding of genes, with the top 100 marker genes per cell type colored by their respective cell types. (**C**) SIMBA–UMAP embedding of peaks, with the top 500 marker peaks per cell type colored by their corresponding cell types. All mappings are conducted in a 50-dimensional space. (**D**) MOMO-GP embedding of cells, (**E**) MOMO-GP embedding of genes, and (**F**) MOMO-GP embedding of peaks, where cells, genes, and peaks are projected into a 2D space. Non-marker genes and peak are shown in gray. For a more quantitative comparison, ACC and ARI values are also presented.

**Figure 7.**
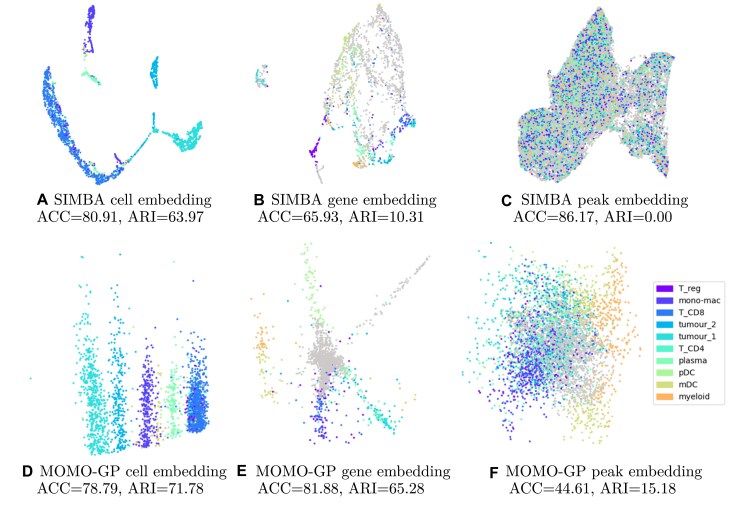
Exploration of the Slide-tag dataset with SIMBA and MOMO-GP techniques applied to both scRNA-seq and scATAC-seq data: (**A**) SIMBA–UMAP embedding of cells color-coded by cell types. (**B**) SIMBA–UMAP embedding of genes, with the top 100 marker genes per cell type colored by their respective cell types. (**C**) SIMBA–UMAP embedding of peaks, with the top 500 marker peaks per cell type colored by their corresponding cell types. All mappings are conducted in a 50-dimensional space. (**D**) MOMO-GP embedding of cells, (**E**) MOMO-GP embedding of genes, and (**F**) MOMO-GP embedding of peaks, where cells, genes, and peaks are projected into a 2D space. Non-marker genes and peak are shown in gray. For a more quantitative comparison, ACC and ARI values are also presented.

In this section, we illustrate the representation of cells using both our method and the SIMBA algorithm. We projected the paired scRNA-seq and scATAC-seq data from the PBMC 10k and Slide-tag datasets, with our MOMO-GP mapping the data into a 2-dimensional space and SIMBA mapping the data into a 50-dimensional (its default value) space. Subsequently, we applied the UMAP method to visualize the results using SIMBA. The findings for PBMC 10k are depicted in Fig. 6A and D and for Slide-tag dataset are shown in Fig. 7A and D. Embedding of cells using PBMC 10k into a 50-dimensional space using MOMO-GP followed by UMAP is given in Supplementary Fig. S11A. By comparing the visualization plots, accuracy, and Rand index values, we observe that there is no significant difference between our method and SIMBA for cell embedding. Both methods achieve a comparable level of performance and effectively separate different cell types. Comparing the results of cell embeddings between the single-view and multi-view approaches, we observe no significant differences in class separation based on the 2D visualizations or the ACC and ARI values. In Supplementary Fig. S12, we show the cell embeddings when we used just scRNA-seq, scATAC-seq data, and also when we used multi-omics data. However, the multi-view approach offers the advantage of generating feature embeddings for all modalities (genes, peaks, and proteins), enabling us to uncover relationships between groups of genes, peaks, or proteins. In the following sections, we will explore these relationships in more detail.

**Figure 8.**
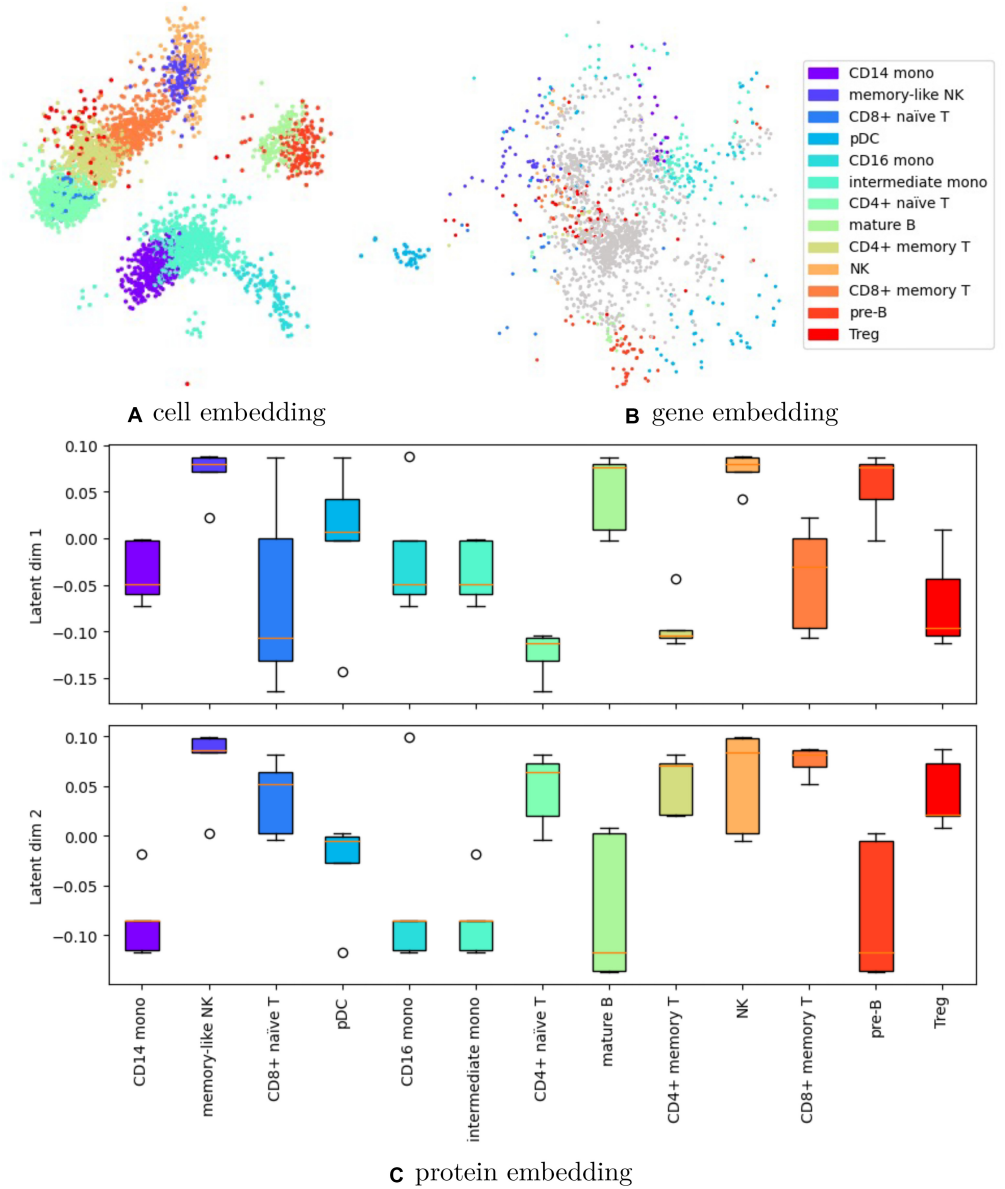
Visualization of MOMO-GP-embedded scRNA-seq data and surface protein data from the PBMC 5k (CITE-seq) dataset, with cells, genes, and proteins mapped to a 2D space. (**A**) Embedding of cells colored by cell types. (**B**) Embedding of genes, with the top 100 marker genes in each cell type colored by their corresponding cell type. Non-marker genes are shown in gray. (**C**) First and second embeddings of proteins, with the top 5 marker proteins considered for each cell type.

Similarly, the cell embedding of MOMO-GP on the PBMC 5k-CITE-seq dataset is presented in Fig. 8A, which shows a good separation between different cell types. We further evaluated multi-view MOMO-GP compared to single-view applications and assessed its robustness to disproportionate feature distributions (e.g. in CITE-seq data). Our analyses indicate that multi-view cell embeddings generally match the quality derived from the most informative single modality (typically RNA-seq) while outperforming sparser modalities. We found that standard MOMO-GP effectively integrates modalities with highly different feature counts (e.g. RNA versus surface markers) without requiring total variance normalization (detailed comparisons are provided in Supplementary Results and  Supplementary Figs S12 and S13). However, as we will discuss in the next subsections, the primary advantage of our model lies in its ability to generate informative feature embeddings, rather than just cell embeddings.

In the multi-view version of MOMO-GP, we utilize a single embedding for the samples and apply the MRD approach to assign distinct coordinates to each view, along with some shared coordinates across both views. To illustrate the shared and specific coordinates, we use scRNA-seq and scATAC-seq data from the PBMC 10k dataset, varying the number of latent dimensions from the set {2, 4, 8, 16, 32}. We present the corresponding ARD values, w_1_ for scRNA-seq data and w_2_ for scATAC-seq data. The results are shown in Supplementary Fig. S14. For each latent dimension, the first bar indicates the ARD values for the scRNA-seq data, while the second bar represents the values for the scATAC-seq data. By analyzing these values and establishing a suitable cutoff, we can identify which dimensions of the cell embedding are specific to the scRNA-seq dataset, which are specific to the scATAC-seq dataset, and which are shared between the two datasets.

In this experiment, when setting the number of latent dimensions to 4, as suggested by the figure, latent variables 3 and 4 are shared between both modalities, latent variable 1 is specific to the scRNA-seq data, and latent variable 2 is absent in both modalities. We provide the cell embedding colored by cell types in Supplementary Fig. S15A for shared coordinates and Supplementary Fig. S15B for specific and absent coordinates. The figure demonstrates a clear separation of cell types for coordinates with high ARD values in both modalities. However, for coordinates with low ARD values in either (RNA or ATAC) or both modalities (absent), this separation of cell types is less distinct.

#### Gene embedding

In this section, we assess the gene embedding produced by the multi-view MOMO-GP model for all PBMC 10k, Slide-tag, and PBMC 5k-CITE-seq datasets. The results for the PBMC 10k dataset are shown in Fig. 6B and E, for Slide-tag data are shown in Fig. 7B and E, while those for the 5k-CITE-seq dataset are presented in Fig. 8B. We conducted a comparison with SIMBA, setting the number of latent dimensions for gene representation to 2 for MOMO-GP and 50 for SIMBA. We visualize the 2D embedding of genes directly obtained from MOMO-GP and the UMAP embedding of SIMBA-embedded data. Embedding of genes into 50-dimensional space using MOMO-GP followed by UMAP is given in Supplementary Fig. S11B. In these figures, we identify the top 100 differentially expressed marker genes and color them according to their respective cell types. The visualization reveals that our gene embedding, using only two latent factors, provides meaningful insights. While there is no perfect separation between marker genes of all cell types, all marker genes of a specific cell type tend to form cohesive clusters. These results highlight the superior performance of our method over SIMBA’s gene embedding.

#### Peak embedding

The peak embeddings generated by both the SIMBA and MOMO-GP models for the PBMC 10k dataset are displayed in Fig. 6C and F, and for Slide-tag dataset are shown in Fig. 7C and F, respectively. In these figures, the number of latent dimensions is set to 2 for MOMO-GP and 50 for SIMBA. The results for 50-dimensional space using MOMO-GP is given in Supplementary Fig. S11C. In these figures, the top 500 marker peaks for each cell type have been selected and colored according to their respective cell types. As observed, our method demonstrates a clear separation between marker peaks, while the results obtained from SIMBA fall short in comparison. It is important to note that in our analysis, we treat the gray values as a separate class. This explains the high accuracy (ACC) observed alongside the very low ARI for SIMBA. In this scenario, the majority cell type in most of the clusters is assigned to the gray values.

#### Protein embedding

The protein embedding generated by our method is depicted in Fig. 8C. Given that the dataset contains a total of 32 proteins, the 2D visualization of data points may not be as informative. Furthermore, due to the overlap between the top 5 marker proteins for different cell types, it was not feasible to color the points based on their relevant cell types. Therefore, we opted to use box plots for this analysis. For each cell type, we selected the top 5 marker proteins and displayed their values for the first and second latent dimensions learned by MOMO-GP. While some overlap exists between relevant cell types such as CD14 mono, CD16 mono, and intermediate mono, there is a clear separation between irrelevant ones, such as B cells and monocyte cells.

#### Interpretability of the model

One of the main advantages of our model is its capability to generate embeddings for both samples and features. In the context of multi-view data analysis, such as CITE-seq data, this translates into distinct embeddings for cells, genes, and proteins. As demonstrated in the single-view version, utilizing the gene relevance map allows us to identify clusters of cells and genes with correlated expression patterns.

Given the abundance of genes in CITE-seq data, aggregating genes into metagenes simplifies analysis and facilitates the assessment of their relevance to meta cells. Similarly, leveraging the protein relevance map enables the identification of clusters of cells and proteins with cohesive expression profiles. With 32 different proteins in CITE-seq data, determining the relevance of each protein to a group of cells becomes straightforward. By identifying cells relevant to each protein and cells relevant to each metagene, we can establish relationships between proteins and genes.

In other types of single-cell omics data, such as scRNA-seq and scATAC-seq, we can similarly uncover relationships between groups of genes and peaks. Overall, the ability of MOMO-GP to generate embeddings for diverse types of data facilitates comprehensive analysis and the discovery of complex relationships between molecular features and cellular phenotypes across various omics datasets.

**Figure 9.**
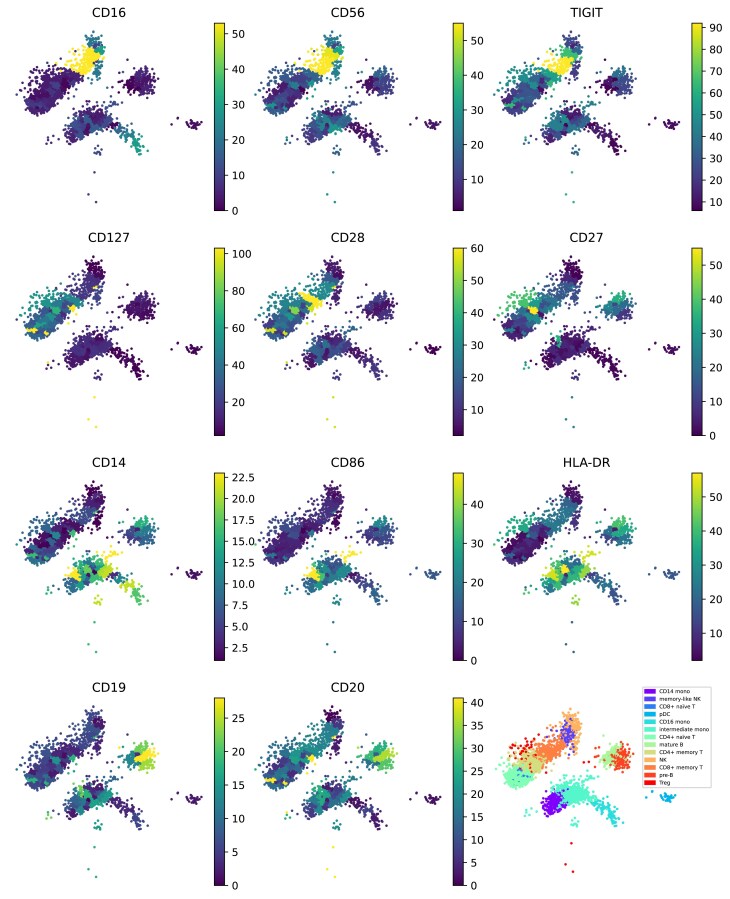
Exploration of the CITE-seq dataset using a protein relevance map, which automatically detects correspondence between groups of cells and proteins. The protein relevance plot highlights areas where the contribution of proteins is highest. For example, in the first row, CD16, CD56, and TIGIT exhibit high relevance in NK and memory-like NK cells. In the second row, CD127, CD28, and CD27 demonstrate high relevance in memory CD4^+^ naïve T, CD8^+^ naïve T, memory CD4^+^ T, and memory CD8^+^ T cells. In the third row, CD14, CD86, and HLA-DR show the highest relevancy in CD14 monocytes, intermediate monocytes, and CD16 monocytes. In the last row, CD19 and CD20 display the highest relevancy in mature B cells and pre-B cells.

Figure 9 illustrates the protein relevance map for the PBMC 5k CITE-seq dataset. Among the 32 proteins analyzed, 11 specific proteins have been identified as highly relevant to distinct cell groups, effectively covering all cells in the dataset. For instance, in the first row of the figure, CD16, CD56, and TIGIT exhibit notable relevance in NK and memory-like NK cells. Moving to the second row, CD127, CD28, and CD27 demonstrate significant relevance in memory CD4^+^ naïve T, CD8^+^ naïve T, memory CD4^+^ T, and memory CD8^+^ T cells. In the third row, CD14, CD86, and HLA-DR exhibit the highest relevancy in CD14 monocytes, intermediate monocytes, and CD16 monocytes. Lastly, in the last row, CD19 and CD20 display the highest relevancy in mature B cells and pre-B cells.

As previously demonstrated in the single-view version, the gene relevance map allows us to determine the relevance of cells and genes. Subsequently, by identifying shared sets of cells relevant to both genes and proteins, we can establish the relevance between genes and proteins. The gene relevance map after running multi-view version of MOMO-GP for the PBMC 5k CITE-seq dataset is shown in Supplementary Fig. S4. In this figure, cells relevant to metagenes 2 and 3 are indicated by circles. The concept of the gene relevance map can be extended to other modalities, allowing for the computation of a protein relevance map, as illustrated in Fig. 9. This figure highlights the relevance of proteins CD16, CD56, and TIGIT to these cells, indicating a potential association between metagenes 2 and 3 and these proteins. This observation is further supported by the fact that these proteins are well-established markers for NK and memory-like NK cells, while the genes associated with metagenes 2 and 3 serve as marker genes for cells of these NK and memory-like NK cell types.

Moreover, Supplementary Fig. S5 presents the gene relevance map for the Slide-tag dataset, where the interpretability features of MOMO-GP are also effective.. The gene relevance map (Supplementary Fig. S5C) successfully links groups of genes (metagenes identified in Supplementary Fig. S5A) to the relevant cell populations in the shared cell embedding. Interestingly, this analysis also provides insights into less characterized gene groups, such as metagene 5 (the gray cluster in Supplementary Fig. S5A), tentatively linking it to monocyte pathways and a specific subset of monocyte/macrophage cells via the relevance map.

### Time complexity of the model

**Figure 10.**
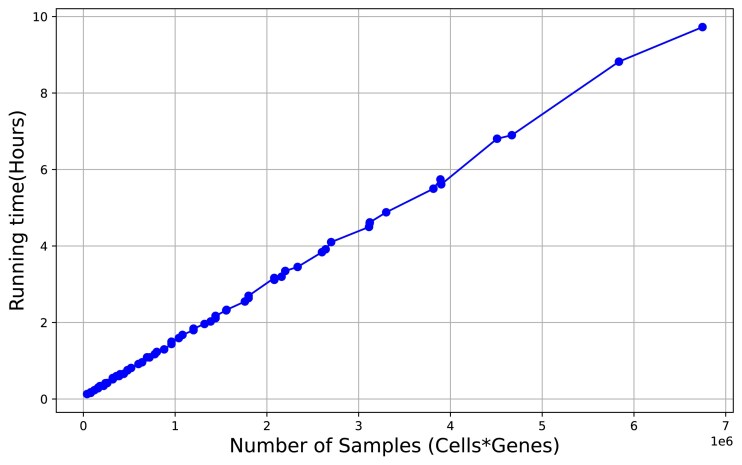
Running time of MOMO-GP by increasing the size of observed data.

The time complexity of our model increases linearly with the number of entities in the observed data. To evaluate this, we tested MOMO-GP on scRNA-seq data from a 5k PBMC CITE-seq dataset. We sampled between 400 and 4000 cells and between 100 and 2000 genes. We then tested all combinations of these cell and gene sets. For each test, we fixed the iteration number at 200, reduced dimensionality to 2, and set the epoch size to 10 000. In this setting, 200 iterations are sufficient for the convergence of the largest dataset. The results are shown in Fig. 10. As demonstrated, increasing the size of the observed data results in a linear increase in the model’s time complexity.

## Discussion

In this section, we discuss the properties of MOMO-GP and potential areas for improvement. MOMO-GP is a novel multi-view latent variable model that captures the nonlinear structure of data by combining a flexible embedding layer with a GP layer. It learns separate latent representations for cells and features (such as genes, peaks, proteins, etc.) in an interpretable manner. By embedding features and utilizing the concept of a gene relevance map, we can identify groups of cells and correlated features from different modalities.

The closest work to ours, which learns sample and feature embeddings, is SIMBA [10].

We compare our results with SIMBA in both single-view and multi-view settings, demonstrating that the feature embeddings learned by our method are more meaningful. In contrast with SIMBA, which uses a single embedding for both samples and features, our method employs different embeddings for each. This enhances the interpretability of the model, as the gene embeddings in MOMO-GP link more naturally to the cell embeddings and perform better than those in SIMBA. We demonstrate this outperformance using various visualization plots and by providing accuracy (ACC) and ARI values.

Proposing a Bayesian version of the model and placing a prior on the latent variables could be explored in future research. Another direction for future work is to place a neural network on top of the embeddings to better capture the nonlinear structure of the data. Considering sample-based data such as time series data or spatial transcriptomic data, where proximity information is crucial, could be another direction for future research. To address this, we should further develop the neural network layer to effectively handle such datasets.

## Conclusion

In this paper, we introduced a new method called MOMO-GP for integrating multi-omics data. The key feature of this method is its ability to simultaneously learn separate feature embeddings for each modality and a shared sample embedding. This approach strikes a balance between expressive power and interpretability. The expressive power is enhanced by linking the embedding layer to the GP layer, while interpretability is achieved by explicitly modeling nonlinear dependencies between features and samples. Through various experiments, we demonstrated that our model outperforms existing algorithms.

## Supplementary Material

gkaf630_Supplemental_Files

## Data Availability

The code for MOMO-GP is available at https://github.com/MLO-lab/MOMO-GP.
