# Supplementary material for: Learning interpretable representations of single-cell multi-omics data with multi-output Gaussian processes
Source: Nucleic Acids Res. 2025 Jul 22;53(14):gkaf630. doi: 10.1093/nar/gkaf630 (PMC12282953; doi:10.1093/nar/gkaf630)
Supplement: gkaf630_Supplemental_Files [file gkaf630_supplemental_files.zip › MOGP_NAR_supplement.pdf]

SUPPLEMENTARY METHODS

Mathematical Foundations of the Single-View MOMO-GP

In this section, we elaborate on the probability distribution and the dependencies between different variables of single-view MOMO-GP. Assuming $\mathbf{Y}\in\mathbb{R}^{I\times J}$ represents the given dataset, where I is the number of data points and J is the number of attributes. In real-world applications, it is common to consider the observed data points $\mathbf{Y}$ as noisy measurements of true values $\mathbf{F}$. This assumption leads to a factorized likelihood given by:

$$p(\mathbf{Y}|\ \mathbf{F},\beta)=\prod_{i=1}^{I}\prod_{j=1}^{J}\mathcal{N}\left(y_{i,j}|f_j(\mathbf{a}_i),\beta^{-1}\right), \tag{3}$$

where $f_j(\mathbf{a}_i)$ represents a function of an r-dimensional latent space. The points in the lower-dimensional latent space are represented by the matrix $\mathbf{A}\in\mathcal{A}=\mathbb{R}^{I\times r_1}$. The relationship between the latent space and the data space is defined by the function $f_j(\mathbf{a}_i)$.

We choose a Gaussian process prior for $\mathbf{F}\in\mathbb{R}^{I\times J}$. Using the idea of GP-LVM, we fit J independent GP regression models on the unobserved latent variable $\mathbf{a}_i$, with

$$p(\mathbf{F}|\mathbf{A})=\prod_{j=1}^{J}\mathcal{N}\left(\mathbf{f}_{:,j}|\mathbf{0},\mathbf{K}_j^{\mathcal{A}}\right), \tag{4}$$

where $\mathbf{K}_j^{\mathcal{A}}\in\mathbb{R}^{I\times I}$ defines the covariance between each pair of $\mathbf{A}$, i.e., $(\mathbf{a}_i,\mathbf{a}_{i'})$. Here, $\mathbf{F}$ is given as the output, and the latent representation $\mathbf{A}$ is optimized.

Now, we deviate from the assumption of independence among J different attributes, recognizing that this assumption is not always accurate. To address this, we introduce a new coregionalization kernel for performing multi-output Gaussian Process regression modeling. This kernel is a separable kernel expressed as the Kronecker product of two individual kernels. The first kernel measures the similarity of samples in the input space, while the second kernel captures the similarity between each pair of features in lower-dimensional space $\mathbf{B}\in\mathcal{B}=\mathbb{R}^{J\times r_2}$, i.e. $(\mathbf{b}_j,\mathbf{b}_{j'})$.

The first kernel, denoted as $\mathbf{K}^{\mathcal{A}}\in\mathbb{R}^{I\times I}$, defines the covariance between each pair of $\mathbf{A}$. Similarly, the second kernel, denoted as $\mathbf{K}^{\mathcal{B}}\in\mathbb{R}^{J\times J}$, defines the covariance between each pair of $\mathbf{B}$. This concept is derived from LVMOGP, where in their supervised scenario, inputs are observed. However, in our model, both kernels act on latent variables $\mathbf{A}$ and $\mathbf{B}$, and they need to be optimized.

The proposed kernel can be expressed as:

$$\mathbf{K}^{\text{coreg}}=\mathbf{K}^{\mathcal{A}}\otimes\mathbf{K}^{\mathcal{B}}, \tag{5}$$

where

$$\begin{cases}\mathbf{K}_{i,i'}^{\mathcal{A}}=k^{\mathcal{A}}(\mathbf{a}_i,\mathbf{a}_{i'})=k^{\mathcal{A}}(i,i'),\\ \mathbf{K}_{j,j'}^{\mathcal{B}}=k^{\mathcal{B}}(\mathbf{b}_j,\mathbf{b}_{j'})=k^{\mathcal{B}}(j,j').\end{cases}$$

In this manner, we consider a correlation between two entities (i,j) and (i',j') in matrix $\mathbf{F}$. This correlation is defined by two kernels over the latent spaces $\mathcal{A}$ and $\mathcal{B}$ as follows:

$$k^{\text{coreg}}((i,j),(i',j'))=k^{\mathcal{A}}(i,i')\cdot k^{\mathcal{B}}(j,j'). \tag{6}$$

The size of this coregionalization kernel is $(I\cdot J)\times(I\cdot J)$ since it computes the correlation for all combinations of I samples and J features in matrix $\mathbf{F}$. Finally, by concatenating all entities of matrix $\mathbf{Y}$, we can model our observed data $(i,j,y_{i,j})$ with a Gaussian Process as follows:

$$p(\mathbf{y})=\mathcal{N}\left(\mathbf{y}|\mathbf{0},\mathbf{K}^{\text{coreg}}+\beta^{-1}\mathbf{I}\right), \tag{7}$$

where $\mathbf{y}\in\mathbb{R}^{I\cdot J}$ is the noisy version of $\mathbf{f}\in\mathbb{R}^{I\cdot J}$ and $\mathbf{K}^{\text{coreg}}\in\mathbb{R}^{(I\cdot J)\times(I\cdot J)}$.

To compute this GP model, we need to compute the inverse of the covariance matrix $\mathbf{K}^{\text{coreg}}$, which has a complexity of $\mathcal{O}(n^3)$, where n is the number of samples, in this case $n=I\cdot J$. In our application, such as gene expression data, we often have a large number of cells (I) and genes (J). To decrease the time complexity of the model and make the problem tractable, we need to employ the idea of sparse GPs.

In sparse GPs, the concept is to expand the probability space with n' different auxiliary pairs of input-output variables collected in matrices $\mathbf{A}_u\in\mathbb{R}^{n'\times r_1}$ and $\mathbf{u}\in\mathbb{R}^{n'\times J}$. Here, the inducing output variables $\mathbf{u}$ are assumed to have the same GP prior as the variables $\mathbf{f}$. The prior over these variables therefore takes the form:

$$p(\mathbf{f},\mathbf{u}|\mathbf{A},\mathbf{A}_u,\boldsymbol{\theta})=\mathcal{N}\left(\begin{bmatrix}\mathbf{f}\\ \mathbf{u}\end{bmatrix}|\mathbf{0},\begin{bmatrix}\mathbf{K}_{ff} & \mathbf{K}_{fu}\\ \mathbf{K}_{uf} & \mathbf{K}_{uu}\end{bmatrix}\right), \tag{8}$$

where $\mathbf{K}_{ff}$ is built by computing the covariance function on all latent variables $\mathbf{A}$, $\mathbf{K}_{uu}$ is constructed by evaluating the covariance function on all auxiliary samples $\mathbf{A}_u$, $\mathbf{K}_{fu}$ is the cross-covariance between latent variables and auxiliary samples, and $\mathbf{K}_{uf}=\mathbf{K}_{fu}^T$. The dependence on variables $\mathbf{A}$, $\mathbf{A}_u$, and the parameters $\boldsymbol{\theta}$ is through these kernel matrices.

The Gaussian process definition allows us to write the marginal distribution and conditional distributions as follows (13):

$$p(\mathbf{u}|\mathbf{A}_u)=\mathcal{N}(\mathbf{u}|\mathbf{0},\mathbf{K}_{uu}), \tag{9}$$

$$\begin{aligned}&p(\mathbf{f}|\mathbf{u},\mathbf{A},\mathbf{A}_u,\boldsymbol{\theta})=\mathcal{N}\left(\mathbf{f}|\mathbf{K}_{fu}\mathbf{K}_{uu}{}^{-1}\mathbf{u},\tilde{\mathbf{K}}\right),\\ &\tilde{\mathbf{K}}=\mathbf{K}_{ff}-\mathbf{K}_{fu}\mathbf{K}_{uu}^{-1}\mathbf{K}_{uf}.\end{aligned} \tag{10}$$

By utilizing equations (9) and (10), the marginal distribution can be expressed as:

$$p(\mathbf{f}|\mathbf{A},\mathbf{A}_u,\boldsymbol{\theta}) = \int p(\mathbf{f}|\mathbf{u},\mathbf{A},\mathbf{A}_u,\boldsymbol{\theta})p(\mathbf{u}|\mathbf{A}_u)d\mathbf{u}$$
$$= \mathcal{N}\left(\mathbf{f}|\mathbf{0},\underbrace{\mathbf{K}_{ff} - \mathbf{K}_{fu}\mathbf{K}_{uu}{}^{-1}\mathbf{K}_{uf}}_{\tilde{\mathbf{K}}} + \mathbf{K}_{fu}\mathbf{K}_{uu}{}^{-1}\mathbf{K}_{uf}\right). \quad (11)$$

Using an approximate posterior, we have:

$$\tilde{p}(\mathbf{f}|\mathbf{u},\mathbf{A},\mathbf{A}_u,\boldsymbol{\theta}) = \mathcal{N}\left(\mathbf{f}|\mathbf{K}_{fu}\mathbf{K}_{uu}{}^{-1}\mathbf{u},\tilde{\mathbf{Q}}\right), \quad (12)$$

where $\tilde{\mathbf{Q}} \neq \tilde{\mathbf{K}}$, and thus the marginal distribution would be:

$$p(\mathbf{f}|\mathbf{A},\mathbf{A}_u,\boldsymbol{\theta}) = \mathcal{N}\left(\mathbf{f}|\mathbf{0},\tilde{\mathbf{Q}} + \mathbf{K}_{fu}\mathbf{K}_{uu}{}^{-1}\mathbf{K}_{uf}\right). \quad (13)$$

There are different kinds of approximation for $\tilde{\mathbf{Q}}$. For example, in conditional (DTC) approximation $\tilde{\mathbf{Q}} = \mathbf{0}$ (40, 41). In our implementation, we use the Scalable Variational Gaussian Process (SVGP) model (42) to speed up the training. These sparse GP models are associated with a computational cost of $O\left(nn'^2\right)$, $n' \ll n$ (42). Decreasing the time complexity is because of avoiding computation and inversion of full covariance matrix $\mathbf{K}_{ff}$, and instead calculation of matrices $\mathbf{K}_{fu}$ and $\mathbf{K}_{uu}$.

In our coregionalization kernel,

$$\mathbf{K}_{uu} = \mathbf{K}^{\mathcal{A}}_{uu} \bigotimes \mathbf{K}^{\mathcal{B}}_{uu}, \quad \text{with} \begin{cases} \mathbf{K}^{\mathcal{A}}_{uu} = \mathbf{K}^{\mathcal{A}}(\mathbf{A}_u,\mathbf{A}_u) \\ \mathbf{K}^{\mathcal{B}}_{uu} = \mathbf{K}^{\mathcal{B}}(\mathbf{B}_u,\mathbf{B}_u) \end{cases}, \quad (14)$$

and

$$\mathbf{K}_{fu} = \mathbf{K}^{\mathcal{A}}_{fu} \bigotimes \mathbf{K}^{\mathcal{B}}_{fu}, \quad \text{with} \begin{cases} \mathbf{K}^{\mathcal{A}}_{fu} = \mathbf{K}^{\mathcal{A}}(\mathbf{A},\mathbf{A}_u) \\ \mathbf{K}^{\mathcal{B}}_{fu} = \mathbf{K}^{\mathcal{B}}(\mathbf{B},\mathbf{B}_u) \end{cases}. \quad (15)$$

In this model, we define m_A inducing points $\mathbf{A}_u$ for the sample space and m_B inducing points $\mathbf{B}_u$ for the feature space. The matrices $\mathbf{K}^{\mathcal{A}}_{uu} \in \mathbb{R}^{m_A \times m_A}$ (resp. $\mathbf{K}^{\mathcal{B}}_{uu} \in \mathbb{R}^{m_B \times m_B}$) compute the similarity between all inducing variables $\mathbf{A}_u$ (resp. $\mathbf{B}_u$) in space $\mathcal{A}$ (resp. in space $\mathcal{B}$), and the matrices $\mathbf{K}^{\mathcal{A}}_{fu} \in \mathbb{R}^{I \times m_A}$ (resp. $\mathbf{K}^{\mathcal{B}}_{fu} \in \mathbb{R}^{J \times m_B}$) compute the cross-covariance between latent variables $\mathbf{A}$ and $\mathbf{A}_u$ (resp. $\mathbf{B}$ and $\mathbf{B}_u$).

By leveraging the concept of sparse GP, we reduce the time complexity of our model from $\mathcal{O}((I \cdot J))^3$ to $\mathcal{O}((I \cdot J) \cdot (m_A \cdot m_B)^2)$. Moreover, we enforce the same number of inducing points for $\mathbf{A}_u$ and $\mathbf{B}_u$ and that allows us to replace the Kronecker product with an elementwise product (18). Using this, we can then further reduce computational complexity to $\mathcal{O}((I \cdot J) \cdot m^2)$).

In our model, the variables that need to be optimized include $\mathbf{A}$, $\mathbf{A}_u$, $\mathbf{B}$, $\mathbf{B}_u$, and other kernel parameters. A novel aspect of our model is the combination of an embedding layer with a Gaussian Process layer to capture the nonlinear structure of the data. Instead of directly optimizing variables $\mathbf{A}$ and $\mathbf{B}$, we utilize an embedding function that converts positive integers (indexes) into dense vectors of fixed size.

To learn $\mathbf{A}$ and $\mathbf{B}$, we map all indices in the range $1,...,I$ and $1,...,J$ to matrices of size $I \times r_1$ and $J \times r_2$, respectively, using an embedding layer. Here, r_1 represents the size of the input embedding space, and r_2 represents the size of the output embedding space. For computing $\mathbf{A}_u$ and $\mathbf{B}_u$, we randomly select from $1,...,I$ and $1,...,J$, respectively, and pass them through the embedding layer to obtain matrices $\mathbf{A}_u$ and $\mathbf{B}_u$ of size $m_A \times r_1$ and $m_B \times r_2$, respectively. During training, the weights of this embedding layer are optimized.

Coupling inducing points We represent the observed data via a triple store where an observed training sample is represented as $(i,j,y_{i,j})$, where $\forall (i,j) \in [1,I] \times [1,J]$ with sample i, feature j and corresponding entries in the observed matrix $y_{i,j}$. In traditional multi-output GP formulations, the covariance matrix of the inducing variables, $\mathbf{K}_{uu}$, is computed as $\mathbf{K}_{uu} = \mathbf{K}^{\mathcal{A}}_{uu} \otimes \mathbf{K}^{\mathcal{B}}_{uu}$. By selecting the same number of inducing points for $\mathbf{A}_u$ and $\mathbf{B}_u$, we can couple the inducing points for both inputs and outputs, allowing us to reformulate the construction of $\mathbf{K}_{uu}$, $\mathbf{K}_{uf}$, and $\mathbf{K}_{fu}$, such that it reflects the coupling between input i and output j of a training sample (i,j). Specifically, we construct the covariance matrix $\mathbf{K}_{uu}$ using the same pairing approach, where the covariance between the o-th and p-th inducing points is computed as:

$$\mathbf{K}_{uu}((\mathbf{A}_u,\mathbf{B}_u),(\mathbf{A}_u,\mathbf{B}_u))_{o,p} =$$
$$k([\mathbf{a}_{u,o},\mathbf{b}_{u,o}],[\mathbf{a}_{u,p},\mathbf{b}_{u,p}]) =$$
$$k^{\mathcal{A}}([\mathbf{a}_{u,o},\mathbf{a}_{u,p}])k^{\mathcal{B}}([\mathbf{b}_{u,o},\mathbf{b}_{u,p}]). \quad (16)$$

Thus, $\mathbf{K}_{uu}$ is expressed as the elementwise product of $\mathbf{K}^{\mathcal{A}}_{uu}$ and $\mathbf{K}^{\mathcal{B}}_{uu}$. This approach reduces the size of $\mathbf{K}_{uu}$ by using an elementwise kernel instead of the Kronecker product. Similarly, the cross-covariance between the q-th training sample (i,j) and the l-th pair of inducing points is as follows:

$$\mathbf{K}_{fu}((\mathbf{A},\mathbf{B}),(\mathbf{A}_u,\mathbf{B}_u))_{q,l} =$$
$$k([\mathbf{a}_i,\mathbf{b}_j],[\mathbf{a}_{u,l},\mathbf{b}_{u,l}]) =$$
$$k^{\mathcal{A}}([\mathbf{a}_i,\mathbf{a}_{u,l}])k^{\mathcal{B}}([\mathbf{b}_j,\mathbf{b}_{u,l}]). \quad (17)$$

This means that by using paired inducing points, $\mathbf{K}_{fu}$ can be written as the elementwise product between $\mathbf{K}^{\mathcal{A}}_{fu}$ and $\mathbf{K}^{\mathcal{B}}_{fu}$. Using this paired formulation,

$$\mathbf{K}_{fu} = \mathbf{K}_{fu}((\mathbf{A},\mathbf{B}),(\mathbf{A}_u,\mathbf{B}_u))$$

becomes smaller in size, resulting in $\mathbf{K}^{\text{paired}}_{fu} \in \mathbb{R}^{(I \cdot J) \times m}$ instead of standard case where we assume independent

Algorithm 1: Single View MOMO-GP (Multi-Omics Multi-Output Gaussian Process algorithm for embedding both samples and features)

Input: $\mathbf{y}$, I, J, r_1, r_2
Output: $\mathbf{A}$, $\mathbf{B}$, $\mathbf{A}_u$, $\mathbf{B}_u$, and kernel parameters $\boldsymbol{\theta}_1$ and $\boldsymbol{\theta}_2$ belong to $k^{\mathcal{A}}$ and $k^{\mathcal{B}}$ kernel functions.
initialize $\mathbf{A}\in\mathbb{R}^{I\times r_1}$, $\mathbf{B}\in\mathbb{R}^{J\times r_2}$, $\mathbf{A}_u\in\mathbb{R}^{m\times r_1}$, $\mathbf{B}_u\in\mathbb{R}^{m\times r_2}$
while *stopping criterion is not satisfied* **do**
  for *each training epoch* **do**
    Calculate the cross-covariance $\mathbf{K}_{fu}^{\text{paired}}\in\mathbb{R}^{(I\cdot J)\times m}$ between latent variables $\mathbf{A}$ and $\mathbf{B}$ and auxiliary samples using Equation (17).
    Calculate the covariance function $\mathbf{K}_{uu}\in\mathbb{R}^{m\times m}$ on all inducing samples $\mathbf{A}_u$ and $\mathbf{B}_u$ using Equation (16).
    Optimize the marginal likelihood distribution $p(\mathbf{y}|\mathbf{A},\mathbf{A}_u,\mathbf{B},\mathbf{B}_u,\boldsymbol{\theta}_1,\boldsymbol{\theta}_2)$ w.r.t. kernel parameters $\boldsymbol{\theta}_1$, $\boldsymbol{\theta}_2$, and $\mathbf{A}$, $\mathbf{B}$, $\mathbf{A}_u$, $\mathbf{B}_u$ by learning weight values of embedding functions in the embedding layer.
  end
end

Algorithm 2: Multi View MOMO-GP (Multi-Omics Multi-Output Gaussian Process algorithm for embedding both samples and features)

Input: $\mathbf{y}_1$,$\mathbf{y}_2$, I, J, K, r_1, r_2, r_3
Output: $\mathbf{A}$, $\mathbf{B}$, $\mathbf{C}$, $\mathbf{A}_u$, $\mathbf{B}_u$, $\mathbf{C}_u$ and kernel parameters $\boldsymbol{\theta}_1$, $\boldsymbol{\theta}_2$, and $\boldsymbol{\theta}_3$ belong to $k^{\mathcal{A}}$, $k^{\mathcal{B}}$, and $k^{\mathcal{C}}$ kernel functions.
initialize $\mathbf{A}\in\mathbb{R}^{I\times r_1}$, $\mathbf{B}\in\mathbb{R}^{J\times r_2}$, $\mathbf{C}\in\mathbb{R}^{K\times r_3}$, $\mathbf{A}_u\in\mathbb{R}^{m\times r_1}$, $\mathbf{B}_u\in\mathbb{R}^{m\times r_2}$,$\mathbf{C}_u\in\mathbb{R}^{m\times r_3}$
while *stopping criterion is not satisfied* **do**
  for *each training epoch* **do**
    Calculate the cross-covariance $\mathbf{K}_{fu}^{1}\in\mathbb{R}^{(I\cdot J)\times m}$ between latent variables $\mathbf{A}$ and $\mathbf{B}$ and auxiliary samples using Equation (22).
    Calculate the covariance function $\mathbf{K}_{uu}^{1}\in\mathbb{R}^{m\times m}$ on all inducing samples $\mathbf{A}_u^1$ and $\mathbf{B}_u$ using Equation (23).
    Calculate the cross-covariance $\mathbf{K}_{fu}^{2}\in\mathbb{R}^{(I\cdot K)\times m}$ between latent variables $\mathbf{A}$ and $\mathbf{C}$ and auxiliary samples using Equation (24).
    Calculate the covariance function $\mathbf{K}_{uu}^{2}\in\mathbb{R}^{m\times m}$ on all inducing samples $\mathbf{A}_u^2$ and $\mathbf{C}_u$ using Equation (25).
    Optimize the marginal likelihood distribution $p\left(\mathbf{y}_1,\mathbf{y}_2|\mathbf{A},\mathbf{A}_u^1,\mathbf{A}_u^2,\mathbf{B},\mathbf{B}_u,\mathbf{C},\mathbf{C}_u,\boldsymbol{\theta}_1,\boldsymbol{\theta}_2,\boldsymbol{\theta}_3\right)$ w.r.t. kernel parameters $\boldsymbol{\theta}_1$, $\boldsymbol{\theta}_2$, $\boldsymbol{\theta}_3$ and $\mathbf{A}$, $\mathbf{B}$, $\mathbf{C}$, $\mathbf{A}_u^1$, $\mathbf{A}_u^2$, $\mathbf{B}_u$, $\mathbf{C}_u$ by learning weight values of embedding functions in the embedding layer.
  end
end

inducing points for input and output resulting $\mathbf{K}_{fu}\in\mathbb{R}^{(I\cdot J)\times(m_A . m_B)}$.

By these formulations, the time complexity of sparse multi-output GP will decrease to $\mathcal{O}((I\cdot J)\cdot m^2)$. Algorithm 1 summarizes all the steps involved in the implementation.

Mathematical Foundations of the Multi-View MOMO-GP

In two view version of MOGP, let I denote the number of samples, J denote the number of features for the first dataset (genes), and K denote the number of features for the second dataset (peaks). $\mathbf{A}\in\mathbb{R}^{I\times r_1}$ represents the low-dimensional embedding of cells, $\mathbf{B}\in\mathbb{R}^{J\times r_2}$ represents the embedding of genes, and $\mathbf{C}\in\mathbb{R}^{K\times r_3}$ represents the embedding of peaks. We define one coregionalization kernel of size $(I\cdot J)\times(I\cdot J)$, formed by the Kronecker product of the covariance matrices $\mathbf{K}^{\mathcal{A}}$ and $\mathbf{K}^{\mathcal{B}}$, and another one of size $(I\cdot K)\times(I\cdot K)$, formed by the Kronecker product of $\mathbf{K}^{\mathcal{A}}$ and $\mathbf{K}^{\mathcal{C}}$. We then define two Gaussian processes, one for generating $\mathbf{f}_1$ using the first kernel and another one for generating $\mathbf{f}_2$ using the second kernel. Using sparse GP for two modalities, we generate two sets of inducing variables, $\mathbf{A}_u^1$, $\mathbf{B}_u$ and $\mathbf{A}_u^2$, $\mathbf{C}_u$. It is important to note that there is no inherent relationship between $\mathbf{A}_u^1$,$\mathbf{B}_u$ from the first modality and $\mathbf{A}_u^2$, $\mathbf{C}_u$ from the second modality. This means that the input and output inducing variables for different modalities are constructed independently. so, our kernels for both modalities are defined as follows:

$$\mathbf{K}_{fu}^1=\mathbf{K}_{fu}^{\mathcal{A}}\bigotimes\mathbf{K}_{fu}^{\mathcal{B}},\quad \text{with}\begin{cases}\mathbf{K}_{fu}^{\mathcal{A}}=\mathbf{K}^{\mathcal{A}}(\mathbf{A},\mathbf{A}_u^1)\\ \mathbf{K}_{fu}^{\mathcal{B}}=\mathbf{K}^{\mathcal{B}}(\mathbf{B},\mathbf{B}_u)\end{cases}, \tag{18}$$

$$\mathbf{K}_{uu}^1=\mathbf{K}_{uu}^{\mathcal{A}}\bigotimes\mathbf{K}_{uu}^{\mathcal{B}},\quad \text{with}\begin{cases}\mathbf{K}_{uu}^{\mathcal{A}}=\mathbf{K}^{\mathcal{A}}(\mathbf{A}_u^1,\mathbf{A}_u^1)\\ \mathbf{K}_{uu}^{\mathcal{B}}=\mathbf{K}^{\mathcal{B}}(\mathbf{B}_u,\mathbf{B}_u)\end{cases}, \tag{19}$$

$$\mathbf{K}_{fu}^2=\mathbf{K}_{fu}^{\mathcal{A}}\bigotimes\mathbf{K}_{fu}^{\mathcal{C}},\quad \text{with}\begin{cases}\mathbf{K}_{fu}^{\mathcal{A}}=\mathbf{K}^{\mathcal{A}}(\mathbf{A},\mathbf{A}_u^2)\\ \mathbf{K}_{fu}^{\mathcal{C}}=\mathbf{K}^{\mathcal{C}}(\mathbf{C},\mathbf{C}_u)\end{cases}. \tag{20}$$

$$\mathbf{K}^2_{uu} = \mathbf{K}^{\mathcal{A}}_{uu} \bigotimes \mathbf{K}^{\mathcal{C}}_{uu}, \quad \text{with} \begin{cases} \mathbf{K}^{\mathcal{A}}_{uu} = \mathbf{K}^{\mathcal{A}}(\mathbf{A}^2_u, \mathbf{A}^2_u) \\ \mathbf{K}^{\mathcal{C}}_{uu} = \mathbf{K}^{\mathcal{C}}(\mathbf{C}_u, \mathbf{C}_u) \end{cases}, \quad (21)$$

However, by coupling the inducing points, they are defined as follows:

$$\mathbf{K}^1_{fu}((\mathbf{A},\mathbf{B}),(\mathbf{A}^1_u,\mathbf{B}_u))_{q,l} = \\ k([\mathbf{a}_i,\mathbf{b}_j],[\mathbf{a}^1_{u,l},\mathbf{b}_{u,l}]) = \\ k_{\mathcal{A}}([\mathbf{a}_i,\mathbf{a}^1_{u,l}])k_{\mathcal{B}}([\mathbf{b}_j,\mathbf{b}_{u,l}]), \quad (22)$$

$$\mathbf{K}^1_{uu}((\mathbf{A}^1_u,\mathbf{B}_u),(\mathbf{A}^1_u,\mathbf{B}_u))_{o,p} = \\ k([\mathbf{a}^1_{u,o},\mathbf{b}_{u,o}],[\mathbf{a}^1_{u,p},\mathbf{b}_{u,p}]) = \\ k_{\mathcal{A}}([\mathbf{a}^1_{u,o},\mathbf{a}^1_{u,p}])k_{\mathcal{B}}([\mathbf{b}_{u,o},\mathbf{b}_{u,p}]), \quad (23)$$

$$\mathbf{K}^2_{fu}((\mathbf{A},\mathbf{C}),(\mathbf{A}^2_u,\mathbf{C}_u))_{q,l} = \\ k([\mathbf{a}_i,\mathbf{c}_j],[\mathbf{a}^2_{u,l},\mathbf{c}_{u,l}]) = \\ k_{\mathcal{A}}([\mathbf{a}_i,\mathbf{a}^2_{u,l}])k_{\mathcal{C}}([\mathbf{c}_j,\mathbf{c}_{u,l}]), \quad (24)$$

$$\mathbf{K}^2_{uu}((\mathbf{A}^2_u,\mathbf{C}_u),(\mathbf{A}^2_u,\mathbf{C}_u))_{o,p} = \\ k([\mathbf{a}^2_{u,o},\mathbf{c}_{u,o}],[\mathbf{a}^2_{u,p},\mathbf{c}_{u,p}]) = \\ k_{\mathcal{A}}([\mathbf{a}^2_{u,o},\mathbf{a}^2_{u,p}])k_{\mathcal{C}}([\mathbf{c}_{u,o},\mathbf{c}_{u,p}]). \quad (25)$$

Finally, to construct the generative model, we optimize the marginal likelihood distribution, as outlined in Algorithm 2 for the two-view version of the method.

Gene relevance maps

The differential d_{gc} for each gene g and cell c describes the change in gene expression space Y along the embedding space A. The partial derivative of d_{gc} for each dimension $r \in \{1,...,R\}$ is defined as follows:

$$d_{gc} = \left(\frac{\partial y_{gc}}{\partial a_{1c}}, ..., \frac{\partial y_{gc}}{\partial a_{Rc}} \right). \quad (26)$$

When computing this derivation is not mathematically possible, we can use the estimation $\widehat{d_{gc}}$ for each cell c using its k nearest neighbors $n \in \text{NN}_k(c)$ as follows:

$$\left(\widehat{d_{gc}}\right)_r = \begin{cases} \text{NA} & \text{if } y_{gc} = 0 \\ \underset{n \in \text{NN}_k(c) \,\&\, n \neq c}{\text{median}} \frac{y_{gc} - y_{gn}}{a_{rc} - a_{rn}} & \text{otherwise} \end{cases} \quad (27)$$

The Euclidean norm of d_{gc} for all dimensions r can then be defined as follows:

$$||d_{gc}||_2 = \sqrt{\sum_{r=1}^{R} (d_{gc})^2_r}. \quad (28)$$

For each cell c, all the genes should be ranked according to the values of $||d_{gc}||_2$ from high to low. If we select a collection of cells $\Psi \subseteq \{1,...,C\}$, define a rank cutoff rg_{max}, and have the rank of genes rg according to the values $||d_{gc}||_2$, then we can define the local gene relevance values for each subset Ψ as follows:

$$\text{LR}_{\text{rg}_{max}}(g,\Psi) = \frac{\sum_{c \in \Psi} [\text{rg} < \text{rg}_{max}]}{|\Psi|}, \quad (29)$$

in which bracket notation

$$[P] = \begin{cases} 1 & \text{if} P \text{is true} \\ 0 & \text{otherwise} \end{cases} \quad (30)$$

The local gene relevance value $\text{LR}(g,\Psi)$ shows if the contribution of gene g in area Ψ is high or not. Global gene relevance $\text{GR}_{\text{rg}_{max}}$ is simply defined by $\text{LR}(g,\Psi)$ if Ψ is the set of all cells:

$$\text{GR}_{\text{rg}_{max}}(g) = \text{LR}_{\text{rg}_{max}}(g,\{1,...,C\}). \quad (31)$$

Finally, a gene relevance map identifies the areas in which the contribution of a subset of genes is highest. These genes can be selected from those with the highest global relevance values.

SUPPLEMENTARY RESULTS

Data pre-processing

To preprocess the PBMC 10k and PBMC 5k (CITE-seq) datasets, we used Scanpy (25) for normalization, logarithmic transformation, clustering, and cluster annotations. Preprocessing of the slide-tag dataset was done using Seurat (26). The specific preprocessing steps for each dataset are described in detail below:

PBMC Data: single-cell RNA-seq. We applied quality control measures to the RNA data, filtering out cells and genes that did not meet predefined quality thresholds based on standard criteria. We filtered cells based on the number of detected genes: cells with fewer genes than a set threshold 200 were considered low quality, while those with more than the threshold 5000 were likely doublets. Next, we filtered cells based on the total RNA molecule count (UMI), keeping only cells with fewer than 15,000 counts. Then, we filter cells with high percentages of mitochondrial gene expression by keeping only cells with less than 20 percent mitochondrial content. Additionally, we excluded genes detected in fewer than a certain number of cells (less than 3 cells), focusing the analysis on more widely

expressed and biologically relevant genes. Subsequently, the data underwent normalization and logarithmic transformation. To annotate cell types, we employed Leiden clustering, and based on the top marker genes of each cluster, we assigned the corresponding cell type. Clusters displaying noise or exhibiting elevated ribosomal gene expression compared to others, or comprising proliferating cells, were excluded. After these procedures, we performed an additional feature selection step to filter genes based on their Coefficient of Variation (CV) relative to their mean expression. This further reduced the number of genes included in our analysis to 2000, ensuring that only the most variable and informative genes were retained for downstream analysis. Finally, we apply z score normalization by subtracting the mean and dividing by the standard deviation to ensure that the data explicitly have a zero mean. This step is essential for our GP model; otherwise, we would need to infer the mean values separately for each modality.

PBMC Data: single-cell ATAC-seq. For the ATAC-seq data, we initially filtered out cells with insufficient peaks and those with peaks detected in too few cells. Specifically, we retained only the peaks present in at least 10 cells for further analysis. Next, we filtered cells based on the number of accessible chromatin regions (peaks) detected per cell, keeping only those with peak counts exceeding 2000 and less than 15,000. Finally, we excluded cells with total counts below 4000 or above 40,000.Regarding normalization, we initially applied the Latent Semantic Indexing (LSI) (43) method, commonly employed in processing ATAC-seq datasets. Subsequently, we applied the same log-normalization procedure utilized in scRNA-seq analysis. Cell type annotation was performed using Leiden clustering, where clusters were annotated based on their marker genes, with some clusters being removed and others annotated accordingly. After these procedures, we conducted an additional feature selection step to filter peaks based on their Coefficient of Variation (CV) in relation to their mean expression. This process further reduced the number of peaks included in our analysis to 5000. Only cells passing the respective quality control criteria were retained in each modality. For integration purposes, only cells present in both modalities were considered.
The number of cells in the intersection of the RNA-seq and ATAC-seq datasets, used in our analysis of the PBMC 10k dataset, amounted to 9393. Finally, similar to scRNA-seq data, we apply z-score normalization to explicitly ensure a zero mean.

PBMC Data: single-cell Protein Data. For the protein expression data, we employed the Denoised and Scaled by Background (DSB) method to normalize and denoise the data from droplet-based single-cell experiments (27). The dataset comprises 32 proteins, and the number of cells in the intersection of the RNA-seq and protein expression data, utilized in our analysis of the 5k PBMCs CITE-seq dataset, amounted to 3891. Finally, z-score normalization must be applied before inputting the data into our GP models.

Slide-tag Data. Log-normalization was applied to both modalities of the dataset. The Slide-tag dataset had already been filtered for low-quality cells, as provided, and therefore no additional cell selection was performed. To identify variable genes, variance stabilizing transformation was used, retaining the 2,000 most variable genes. Similarly, the 5,000 most variable peaks were selected based on the highest variance. Finally, z-score normalization was applied before running our GP models. Cell-type annotations were provided with the data set.

Evaluation metrics

Rand Index and Adjusted Rand Index. The Rand Index computes the similarity between two different clusterings. We should count all pairs of data points that are from the same cluster or different clusters in two different clusterings. Given a set S of n different elements and two partitions of S, $X=\{X_1,...,X_r\}$, a partition of S into r subsets, and $Y=\{Y_1,...,Y_s\}$, a partition of S into s subsets. Then, we define:
- a as the number of pairs of elements in S which are in the same cluster in X and in the same cluster in Y,
- b as the number of pairs of elements which are in different clusters in X and in different clusters in Y,
- c as the number of pairs of elements which are in the same cluster in X and in different clusters in Y,
- d as the number of pairs of elements which are in different clusters in X and in the same cluster in Y.

Using these notations, the Rand Index is calculated as follows:

$$\mathrm{RI}=\frac{a+b}{a+b+c+d}. \tag{32}$$

The values of the Rand Index range from 0 to 1, where a value of 0 indicates that the output of both clusterings is completely different, and a value of 1 indicates that both clusterings create exactly the same result.

The Adjusted Rand Index is an extension of the Rand Index. It ranges from -1 to 1, where a value of 1 indicates identical clusterings, a value of 0 indicates random clusterings, and a value of -1 indicates complete disagreement between clusterings. The ARI formula is as follows:

$$\mathrm{ARI}=\frac{\text{RI - Expected\_RI}}{\text{max(RI) - Expected\_RI}}, \tag{33}$$

where Expected_RI is the expected value of the Rand Index using the permutation model for clusterings.

Comparison of different metrics. We used both Accuracy (ACC) and Adjusted Rand Index (ARI) to provide a comprehensive evaluation of our clustering performance, as each metric captures different aspects of clustering quality. Accuracy measures how well the predicted clusters match the ground truth labels, making it an intuitive and straightforward metric. However, accuracy alone can be misleading, particularly in imbalanced datasets, where a model may achieve high accuracy simply by assigning most samples to the dominant class while failing to correctly classify minority classes. ARI, on the other hand, assesses the similarity between predicted clusters and true labels by considering how well pairs of samples are grouped together. Unlike accuracy, ARI is adjusted for chance and is not influenced by class distribution, making it a more reliable

indicator of clustering quality. A model with high accuracy but low ARI may correctly classify a large number of samples but fail to maintain the correct clustering structure. Conversely, a high ARI with lower accuracy suggests that the clusters are well-formed but may not perfectly align with the ground truth labels. By considering both ACC and ARI, we ensure that our evaluation captures both absolute correctness (the proportion of correctly assigned labels) and structural validity (how well the clustering reflects the true structure of the data). This dual approach prevents misleading conclusions and provides a more balanced, insightful assessment of our model's performance. Moreover, to ensure more robust and comprehensive evaluations, we have included additional performance metrics. Specifically, we report results for silhouette, NMI, and isolated labels, each of which provides valuable insights into different aspects of the model's performance: Silhouette score evaluates the clustering quality by measuring how well-separated clusters are, with higher values indicating better-defined clusters. NMI (Normalized Mutual Information) quantifies the mutual dependence between predicted clusters and true labels, with higher values representing better clustering performance. Isolated labels score evaluates the separation of distinct cell populations, where higher values indicate better identification of isolated clusters.

Multi-view vs single-view embeddings

To demonstrate that our method effectively utilizes information from the second modality, even with a limited number of features (32 proteins), we present cell embeddings generated using only protein data. The results, shown in Figure S13, indicate that despite relying on a single modality, the separation between different cell types remains relatively preserved. Additionally, this figure presents cell embeddings obtained after scaling each modality to ensure a total variance of 1. This normalization balances the influence of scRNA-seq data, which has significantly more features and higher variance than other modalities, preventing any single modality from dominating the analysis. We compare four scenarios: embeddings generated using only scRNA-seq data, only protein data, unscaled multi-view data, and scaled multi-view data with total variance normalized to 1. The results indicate that embeddings generated using only protein data yield the weakest performance. Similarly, applying total variance normalization to 1 leads to suboptimal results, as this approach overemphasizes protein data while reducing the contribution of RNA data. In contrast, the best results are achieved when using only RNA data, followed by multi-view data without variance scaling.

SUPPLEMENTARY FIGURES

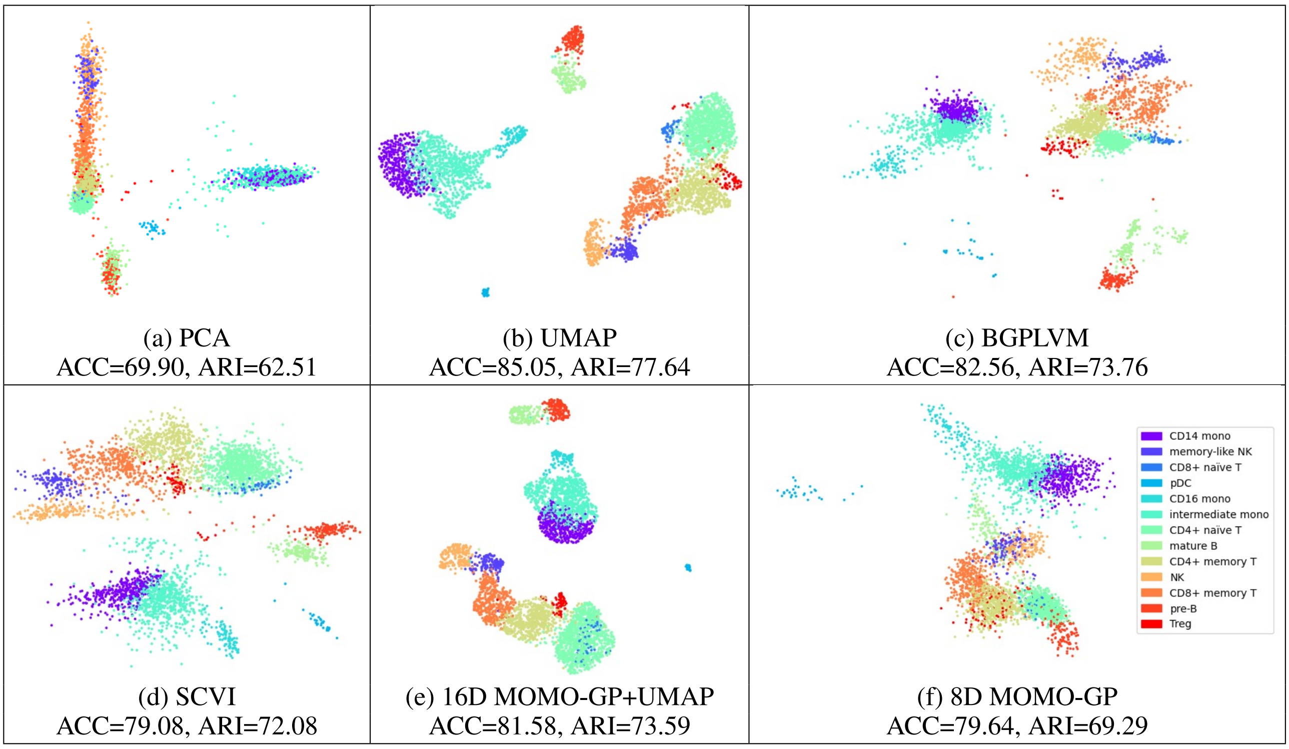

Figure S1. 2D visualization of cells in PBMC 5k (CITE-seq) dataset for scRNA-seq data using various methods: (a) PCA, (b) UMAP, (c) BGPLVM, (d) SCVI, (e) 16D MOMO-GP+UMAP, and (f) 8D MOMO-GP+UMAP.

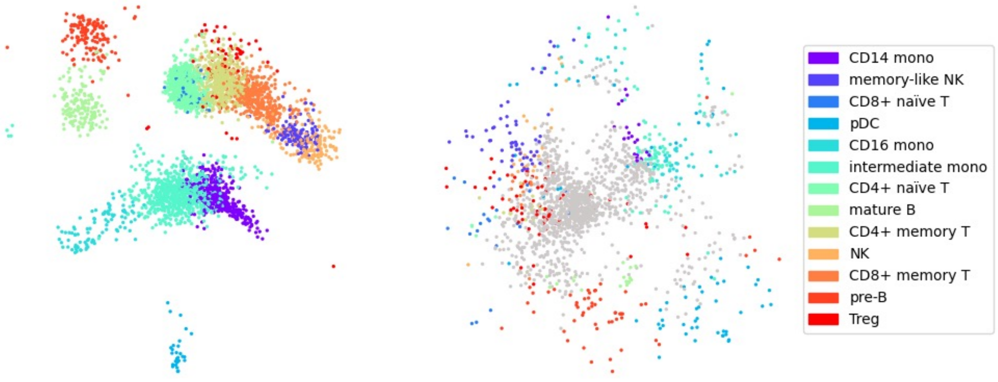

(a) cell embedding
ACC=80.51, ARI=70.97

(b) gene embedding
ACC=74.40, ARI=28.07

Figure S2. Visualization of MOMO-GP-embedded scRNA-seq data from the PBMC 5k (CITE-seq) dataset, where both cells and genes are mapped to a 2D space: (a) Embedding of cells colored by cell types, (b) Embedding of genes with the top 100 marker genes in each cell type colored by their corresponding cell type. Non-marker genes are shown in gray.

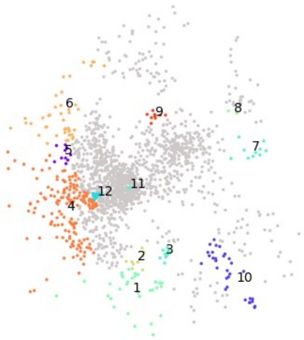

(a) meta genes and their associated genes

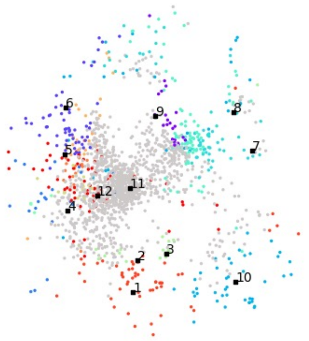

(b) marker genes

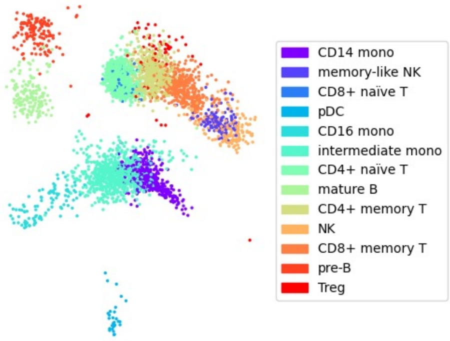

(c) cell embedding

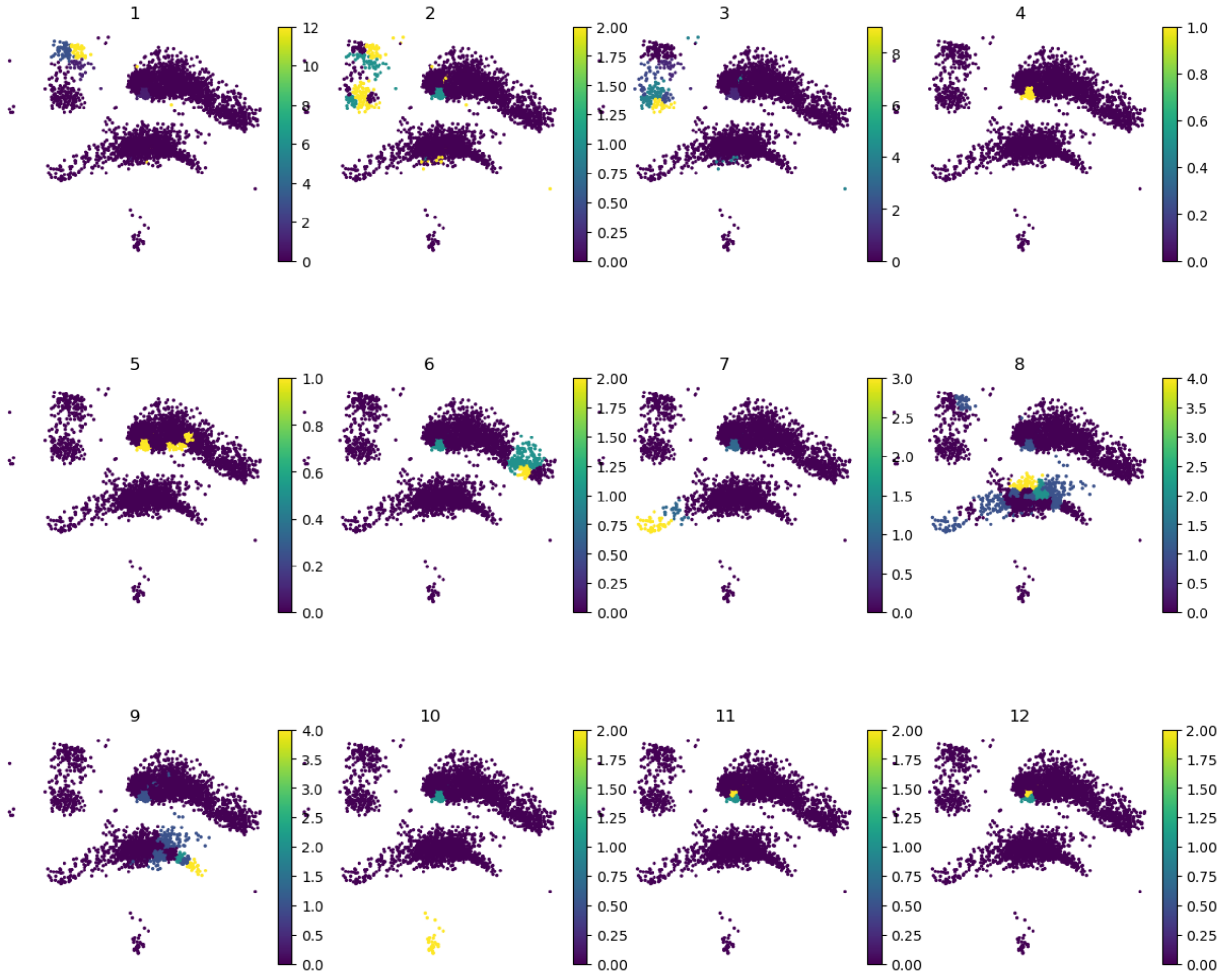

(d) highest relevant meta genes

Figure S3. Exploration of the PBMC 5k (CITE-seq) dataset (single-view version) using a gene relevance map, which automatically detects correspondence between groups of cells and genes: (a) Gene embedding colored by genes associated with each meta-gene. (b) Gene embedding colored by marker genes specific to each cell type. (c) Cell embedding colored by cell types. (d) Gene relevance plot highlighting areas where the contribution of genes is highest. For example, meta-gene 10 exhibits high relevance in the lower region of the cell embedding.

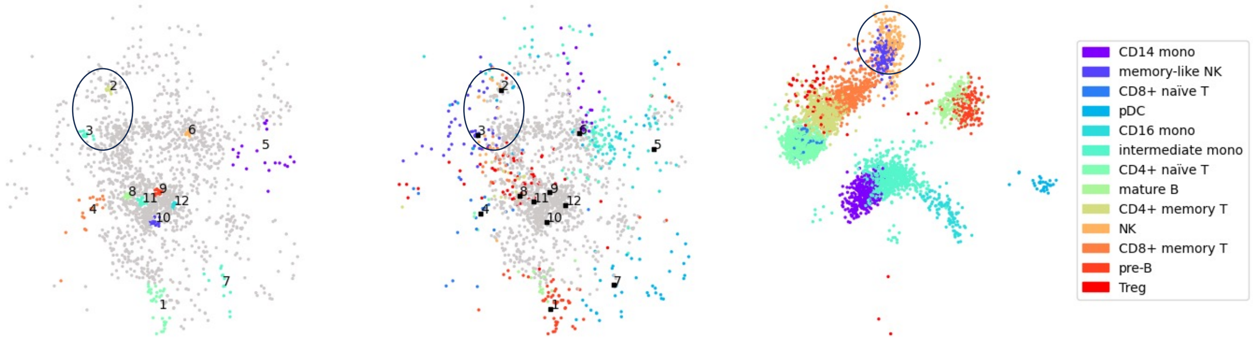

(a) meta genes and their associated genes (b) marker genes (c) cell types

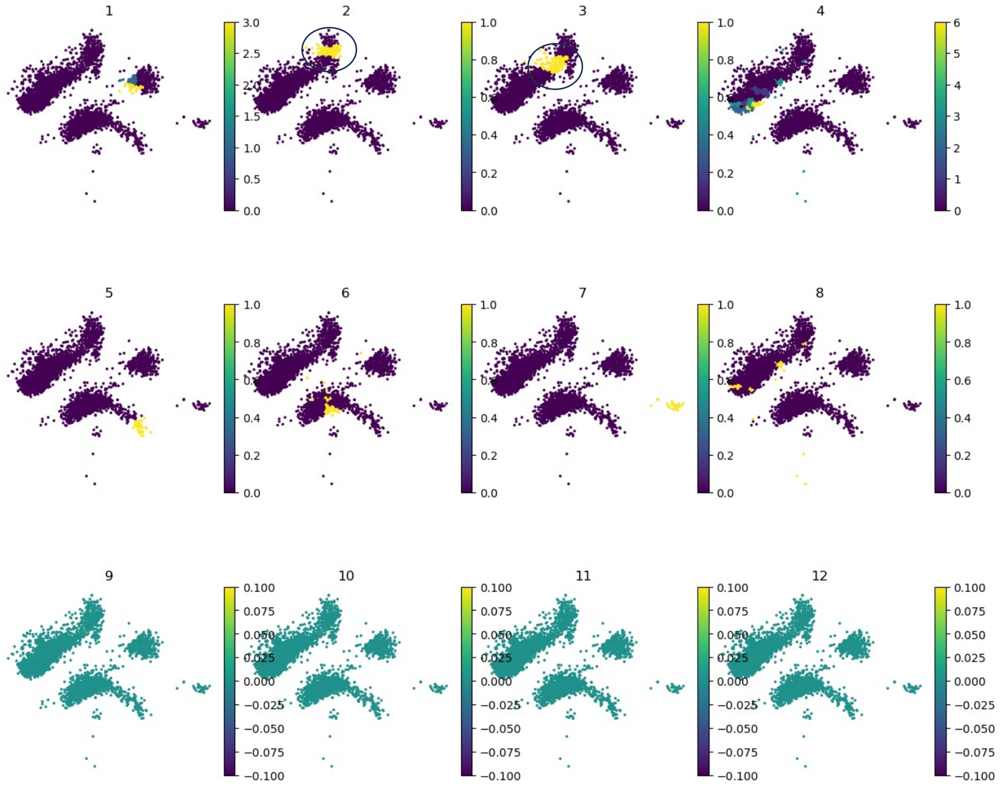

(d) highest relevant meta genes

Figure S4. Exploration of the PBMC 5k (CITE-seq) dataset (multi-view version) using a gene relevance map, which automatically detects correspondence between groups of cells and genes: (a) Gene embedding colored by genes associated with each meta-gene. (b) Gene embedding colored by marker genes specific to each cell type. (c) Cell embedding colored by cell types. (d) Gene relevance plot highlighting areas where the contribution of genes is highest. For example, genes associated with meta-genes 2 and 3 are highly relevant to cells classified as NK and memory-like NK cell types, as indicated by the circles.

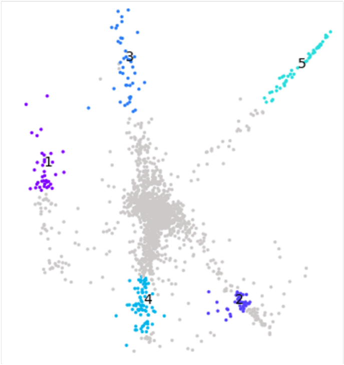

(a) meta genes and their associated genes

Meta Gene	Term	Adjusted P-value	Combined Score
1	Stromal	2.58×10^{-3}	61.57
1	Erythroblast	0.370	5.03
2	Eosinophil	4.16×10^{-3}	268.40
2	Dendritic	7.18×10^{-7}	256.07
3	Plasma	4.19×10^{-15}	1113.48
3	CD34 POS HSC	0.385	12.32
4	CD8 T	5.68×10^{-10}	400.32
4	Naive T	7.92×10^{-12}	371.88
5	Platelet	0.370	4.79
5	Monocyte	0.421	3.18

(b) enriched gene sets for each meta-gene

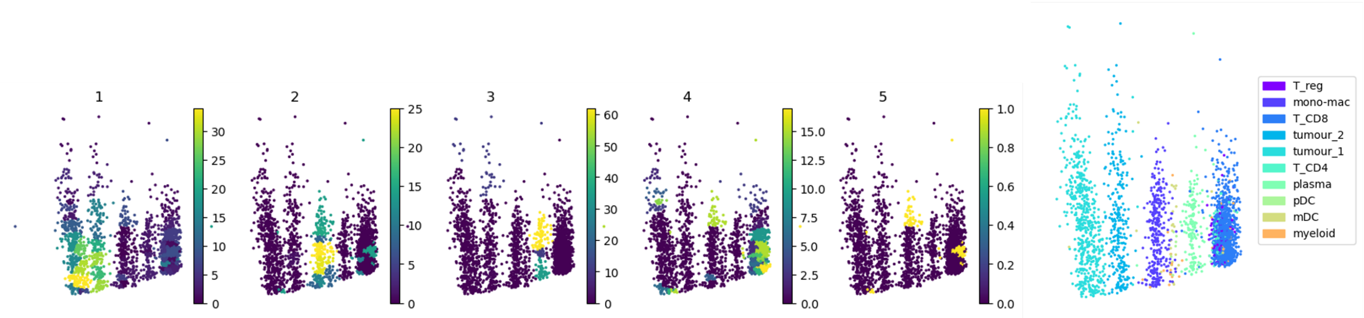

(c) highest relevant meta genes and cell types in the embedding space

Figure S5. Exploration of the Slide Tag dataset using a gene relevance map, which automatically detects correspondence between groups of cells and genes: (a) Gene embedding colored by genes associated with each meta-gene. (b) List of gene sets enriched for each meta-gene. (c) Gene relevance plot highlighting areas where the contribution of genes is highest and cell embedding colored by cell types.

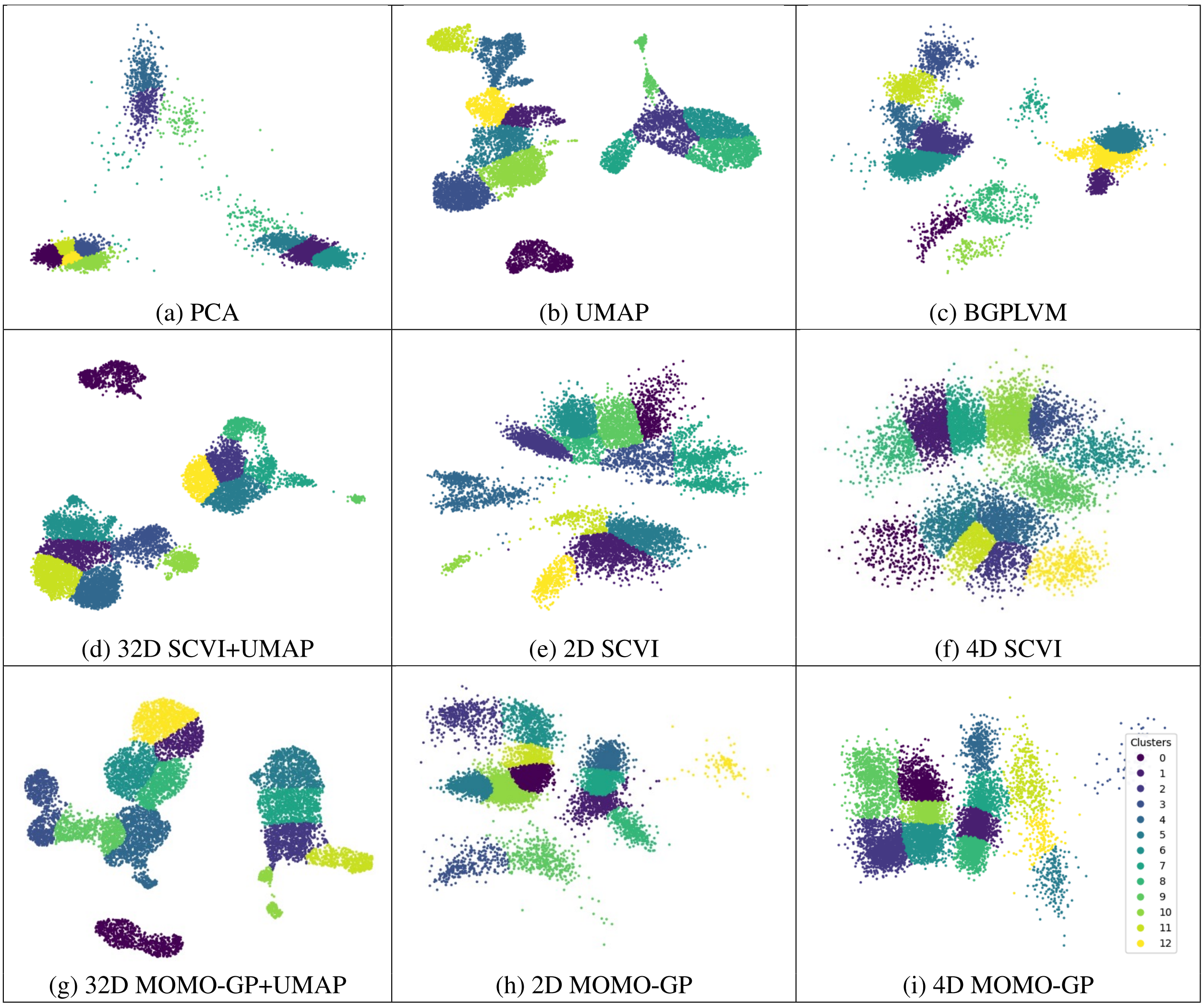

Figure S6. 2D visualization of cells colored by GMM clusters in the PBMC 10k dataset using various methods: (a) 2D PCA, (b) 2D UMAP, (c) 2D BGPLVM, (d) 32D SCVI+UMAP, (e) 2D SCVI, (f) 4D SCVI, (g) 32D MOMO-GP+UMAP, (h) 2D MOMO-GP, and (i) 4D MOMO-GP.

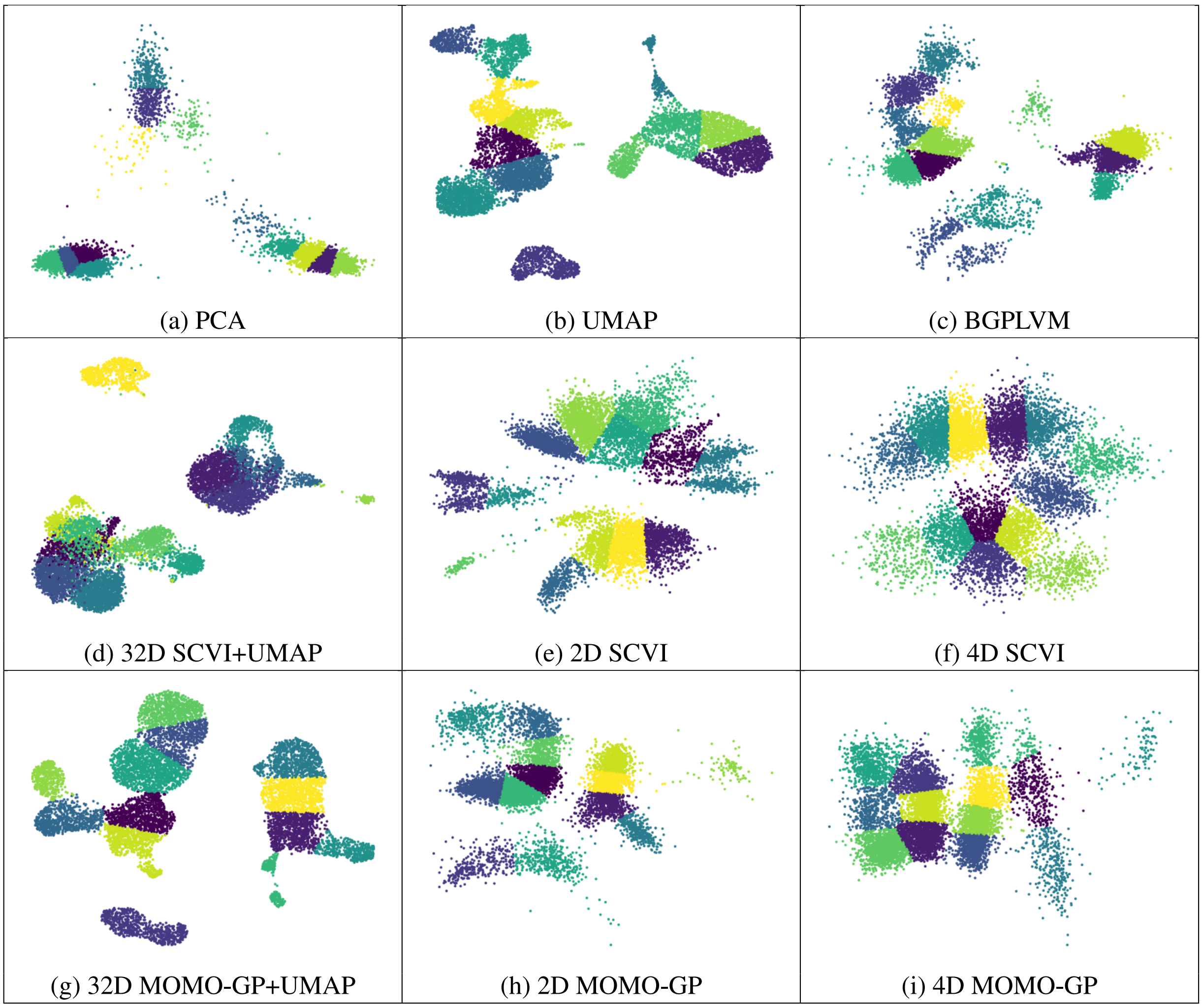

Figure S7. 2D visualization of cells colored by K-Means clusters in the PBMC 10k dataset using various methods: (a) 2D PCA, (b) 2D UMAP, (c) 2D BGPLVM, (d) 32D SCVI+UMAP, (e) 2D SCVI, (f) 4D SCVI, (g) 32D MOMO-GP+UMAP, (h) 2D MOMO-GP, and (i) 4D MOMO-GP.

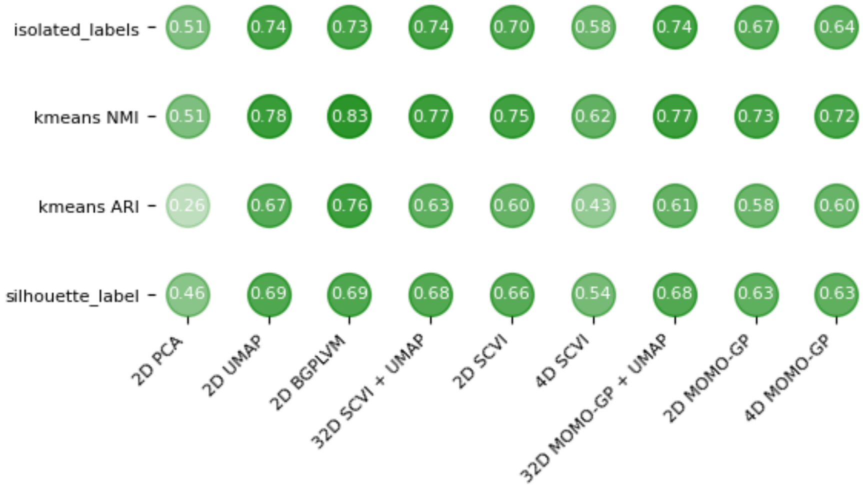

Figure S8. Analysing scRNA-seq data from PBMC 10k dataset by comparison of various methods 2D PCA, 2D UMAP, 2D BGPLVM, 32D SCVI+UMAP, 2D SCVI, 4D SCVI, 32D MOMO-GP+UMAP, 2D MOMO-GP, and 4D MOMO-GP using different metrics.

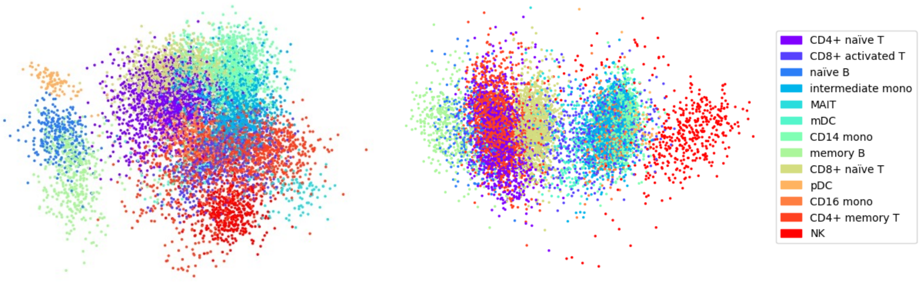

(a) SCVI
ACC=53.11, ARI=29.52

(b) MOMO-GP
ACC=49.78, ARI=33.56

Figure S9. 2D visualization of cells in the PBMC 10k dataset for scRNA-seq data using: a) 4D SCVI, and b) 4D MOMO-GP.

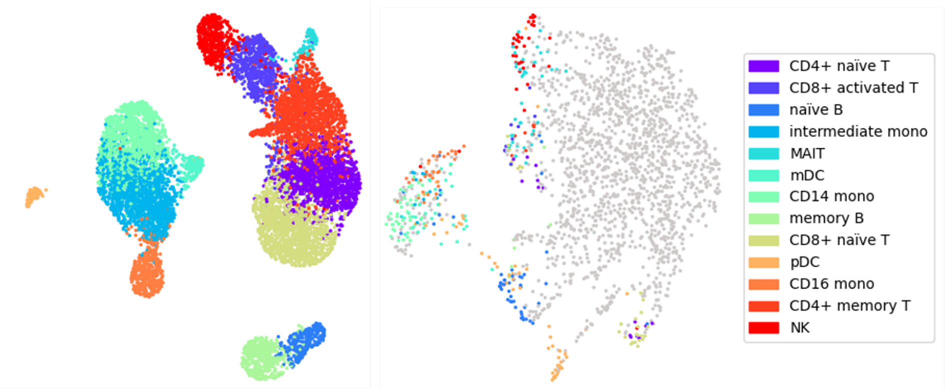

(a) cell embedding
ACC=81.71, ARI=67.95

(b) gene embedding
ACC=82.6, ARI=39.28

Figure S10. Visualization of PBMC 10k dataset using MOMO-GP embedding techniques for scRNA-seq data. (a) MOMO-GP-UMAP embedding of cells, with cell types color-coded, in a 50-D space. (b) MOMO-GP-UMAP embedding of genes, highlighting the top 100 marker genes per cell type, color-coded by their respective cell types, in a 50-D space. Non-marker genes are shown in gray.

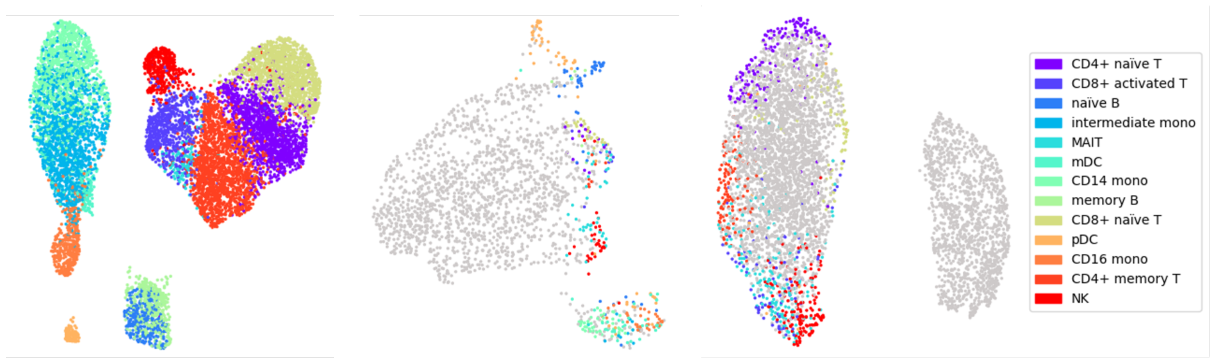

(a) MOMO-GP cell embedding
ACC=77.81, ARI=63.78

(b) MOMO-GP gene embedding
ACC=82.69, ARI=32.11

(c) MOMO-GP peak embedding
ACC=84.45, ARI=9.94

Figure S11. Exploration of the PBMC 10k dataset with MOMO-GP embedding techniques applied to both scRNA-seq and scATAC-seq data: (a) MOMO-GP embedding of cells, (b) MOMO-GP embedding of genes, and (c) MOMO-GP embedding of peaks, where cells, genes, and peaks are projected into a 50-dimensional space using MOMO-GP followed by UMAP. Non-marker genes and peak are shown in gray.

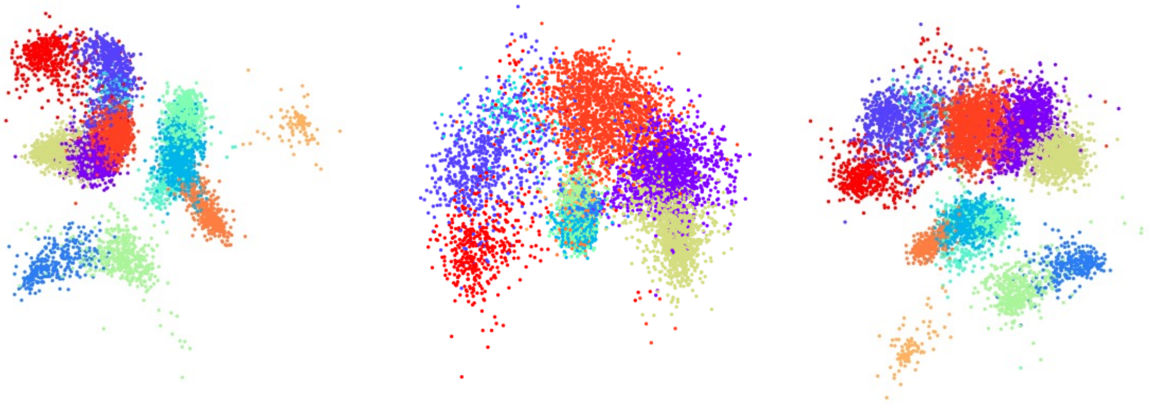

(a) scRNA-seq data
ACC=79.85, ARI=61.17

(b) scATAC-seq data
ACC=64.88, ARI=51.52

(c) Multi-Omic data
ACC=75.01, ARI=63.14

Figure S12. Comparison of MOMO-GP cell embeddings using scRNA-seq data, scATAC-seq data, and the multi-view MOMO-GP approach with multi-omic data from the PBMC 10k dataset.

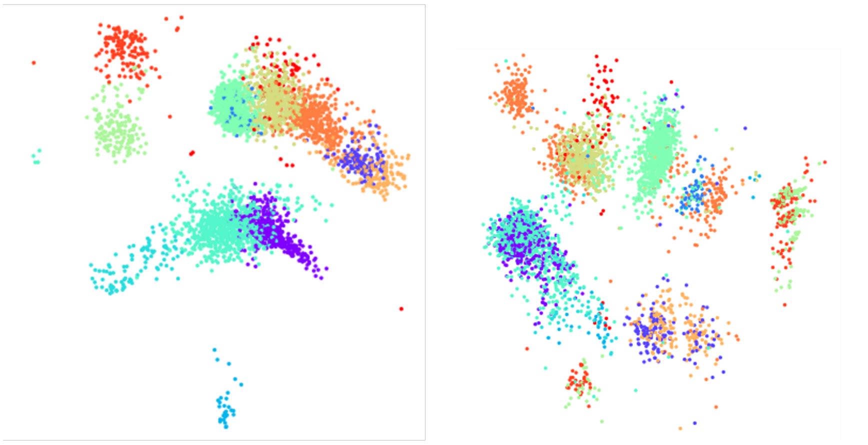

(a) ACC=80.51, ARI=70.97

(b) ACC=67.78, ARI=60.42

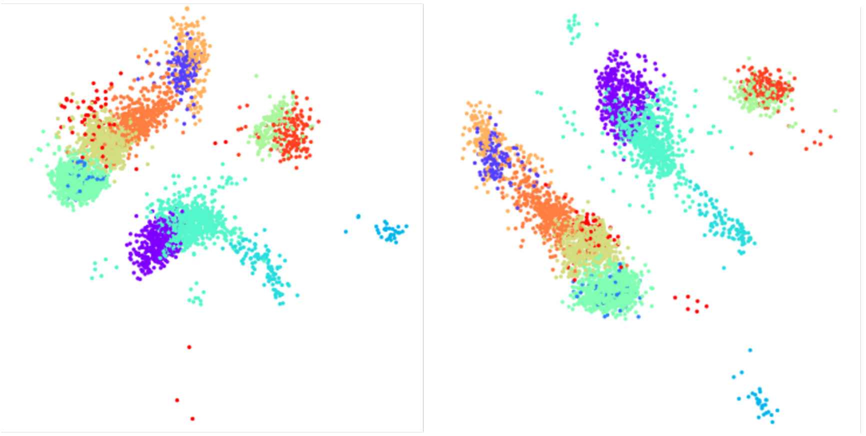

(c) ACC=78.40, ARI=71.73

(d) ACC=75.84, ARI=69.13

Figure S13. Visualization of MOMO-GP-embedded data from the PBMC 5k (CITE-seq) dataset, with cells mapped to a 2D space colored by cell types using (a) scRNA-seq data, (b) protein data, (c) multi-view data, and (d) multi-view data after scaling each data modality to have a total variance of 1.

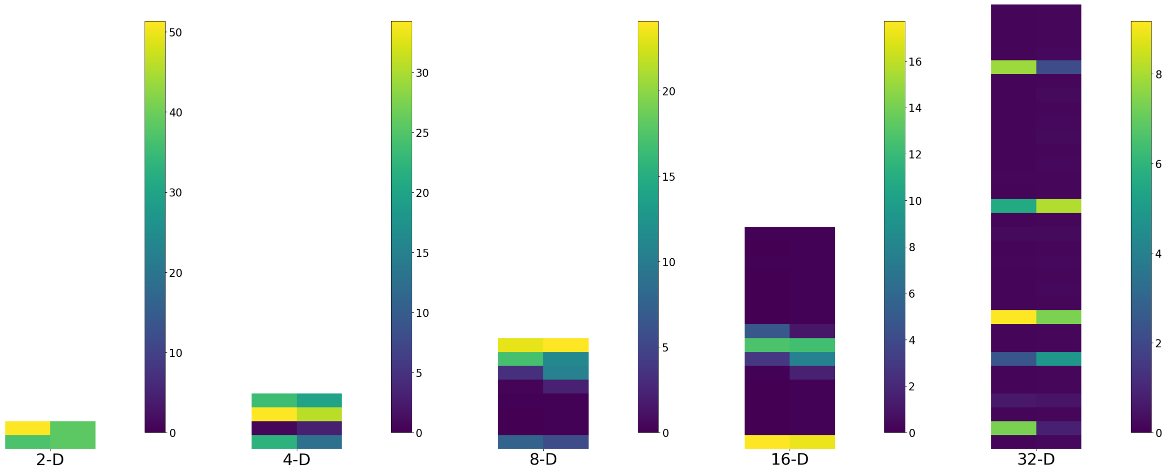

Figure S14. The corresponding ARD values, $\mathbf{w}_1$ for the scRNA-seq data and $\mathbf{w}_2$ for the scATAC-seq data from PBMC 10k dataset. We change the values of different latent dimensions from the set $\{2, 4, 8, 16, 32\}$. For each latent dimension, the first bar represents the ARD values for the scRNA-seq data, while the second bar represents the values for the scATAC-seq data. By comparing these values, we can determine which dimensions of the cell embedding are specific to the scRNA-seq dataset, which are specific to the scATAC-seq dataset, and which are shared between both datasets.

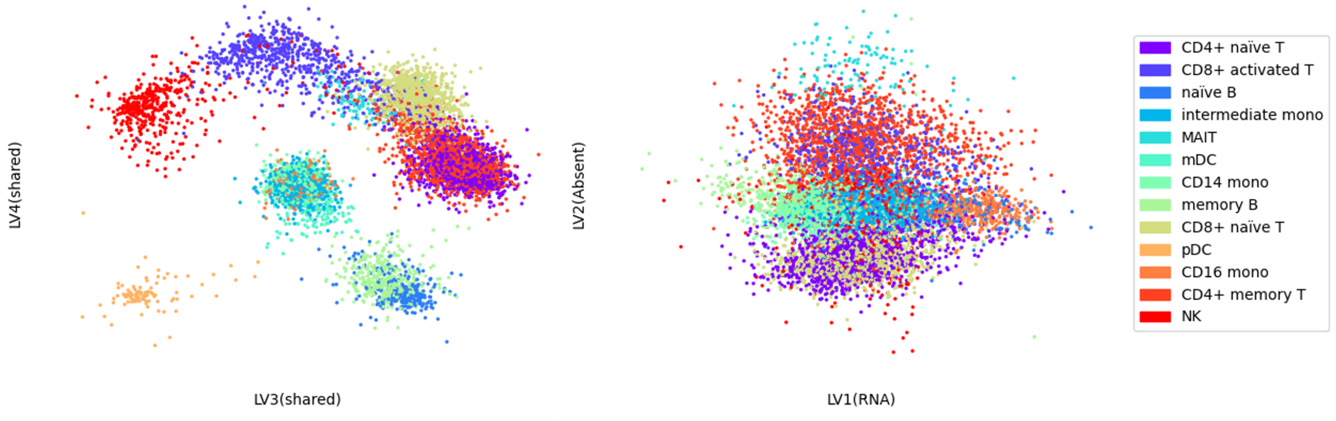

(a) (b)

Figure S15. MOMO-GP embedding of cells from the PBMC 10k dataset applied to both scRNA-seq and scATAC-seq data: (a) cell embedding with shared latent variables, (b) cell embedding with specific and absent latent variables.

SUPPLEMENTARY TABLES

Table S1. PBMC 10k dataset (scRNA-seq data): The list of top 20 genes located near the center of data embedded by MOMO-GP into 2D space.

The list of top 20 genes				
IGHV3-66	AC022445.1	DZIP1	AC005481.1	AC005481.1
AC024933.1	LINC02821	MEIOB	ERVK-28	CARMN
HEPHL1	AL590999.1	MS4A3	LINGO4	HSPB8
AC079035.1	DNTT	AL589740.1	AC092134.1	HABP2

Table S2. PBMC 5k (CITE-seq) dataset: A list of gene sets enriched for each meta-gene.

Meta Gene	Term	Adjusted P-value	Combined Score	Cell-type Coverage
1	Follicular B	6.47×10^{-15}	926.91	81.13
2	Follicular B	1.30×10^{-8}	1829.58	77.35
3	Plasma	6.76×10^{-3}	302.32	NA
3	Follicular B	1.01×10^{-2}	129.68	100
4	Naïve T	9.71×10^{-82}	3120.82	99.58
5	NK	8.40×10^{-5}	280.38	0.00
6	NK	4.77×10^{-30}	3560.08	100
7	Monocyte	4.72×10^{-10}	3685.82	100
9	Neutrophil	6.40×10^{-5}	382.07	NA
10	Dendritic	4.49×10^{-16}	1289.41	100
