# Supplementary material for: Learning interpretable representations of single-cell multi-omics data with multi-output Gaussian processes
Source: Nucleic Acids Res. 2025 Jul 22;53(14):gkaf630. doi: 10.1093/nar/gkaf630 (PMC12282953; doi:10.1093/nar/gkaf630)
Supplement: gkaf630_Supplemental_Files [file gkaf630_supplemental_files.zip › MOMO-GP-code.zip!/MOMO-GP-main/experiments/CITEseq/RNA/Timing/RunningTime.pdf]

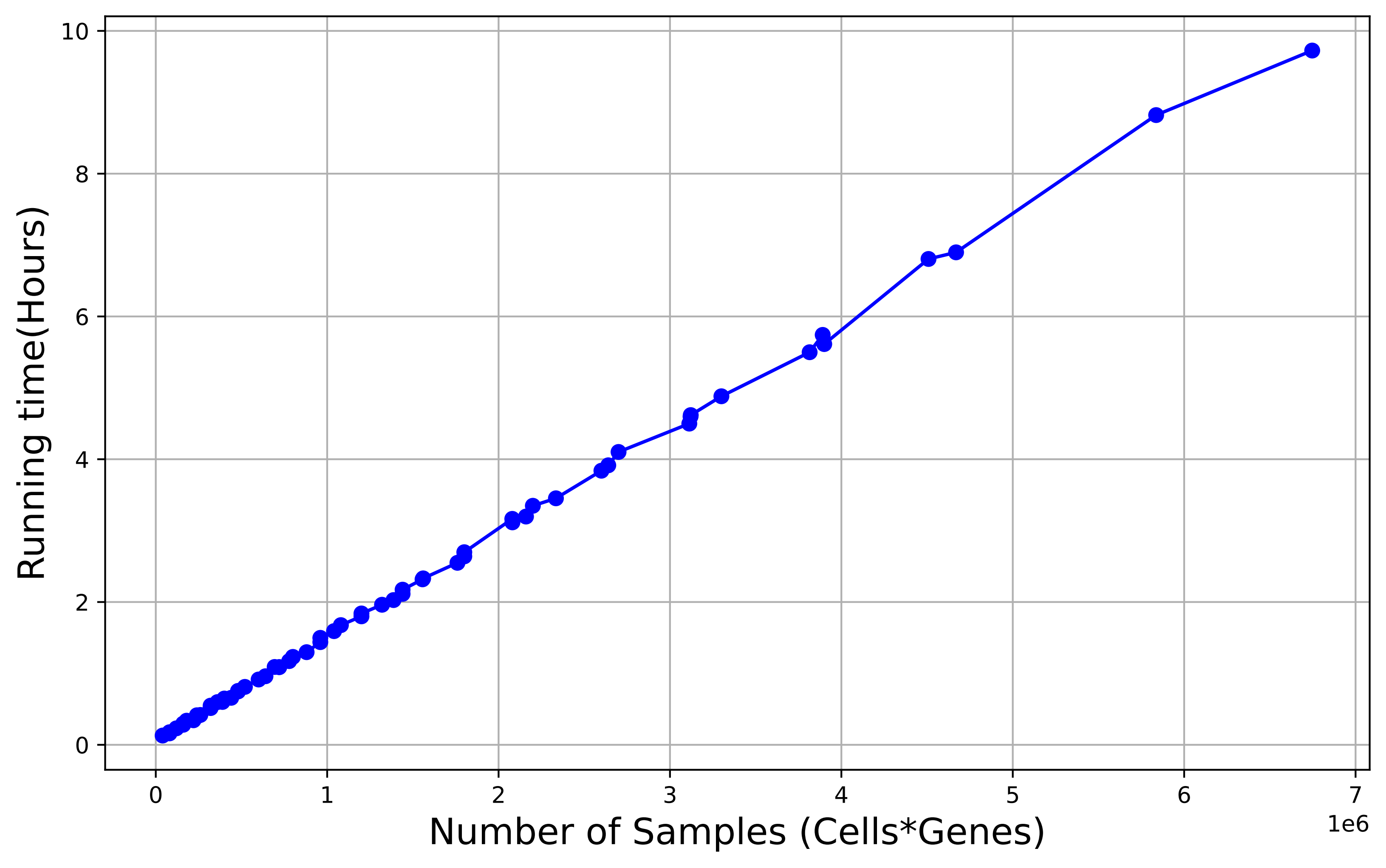
Running time(Hours)
0
2
4
6
8
10
0
1
2
3
4
5
6
7
1e6
Number of Samples (Cells*Genes)
